# Vibrational energy transfer in collisions of molecules with metal surfaces

**DOI:** 10.1039/d4cp00957f

**Published:** 2024-04-29

**Authors:** Igor Rahinov, Alexander Kandratsenka, Tim Schäfer, Pranav Shirhatti, Kai Golibrzuch, Alec M. Wodtke

**Affiliations:** a Department of Natural Sciences, The Open University of Israel 4353701 Raanana Israel igorra@openu.ac.il; b Department of Dynamics at Surfaces, Max Planck Institute for Multidisciplinary Sciences Am Fassberg 11 37077 Goettingen Germany alec.wodtke@mpinat.mpg.de; c Institute for Physical Chemistry, Georg-August University of Goettingen Tammannstraße 6 37077 Goettingen Germany; d Tata Institute of Fundamental Research Hyderabad 36/P Gopanpally Hyderabad 500046 Telangana India; e International Center for Advanced Studies of Energy Conversion, Georg-August University of Goettingen Tammannstraße 6 37077 Goettingen Germany

## Abstract

The Born–Oppenheimer approximation (BOA), which serves as the basis for our understanding of chemical bonding, reactivity and dynamics, is routinely violated for vibrationally inelastic scattering of molecules at metal surfaces. The title-field therefore represents a fascinating challenge to our conventional wisdom calling for new concepts that involve explicit electron dynamics occurring in concert with nuclear motion. Here, we review progress made in this field over the last decade, which has witnessed dramatic advances in experimental methods, thereby providing a much more extensive set of diverse observations than has ever before been available. We first review the experimental methods used in this field and then provide a systematic tour of the vast array of observations that are currently available. We show how these observations – taken together and without reference to computational simulations – lead us to a simple and intuitive picture of BOA failure in molecular dynamics at metal surfaces, one where electron transfer between the molecule and the metal plays a preeminent role. We also review recent progress made in the theory of electron transfer mediated BOA failure in molecule–surface interactions, describing the most important methods and their ability to reproduce experimental observation. Finally, we outline future directions for research and important unanswered questions.

## Abstract 2

A systematic perspective of the progress in observations and theory of electronically nonadiabatic vibrational energy transfer during molecule–metal surface collisions.

## Graphical abstract

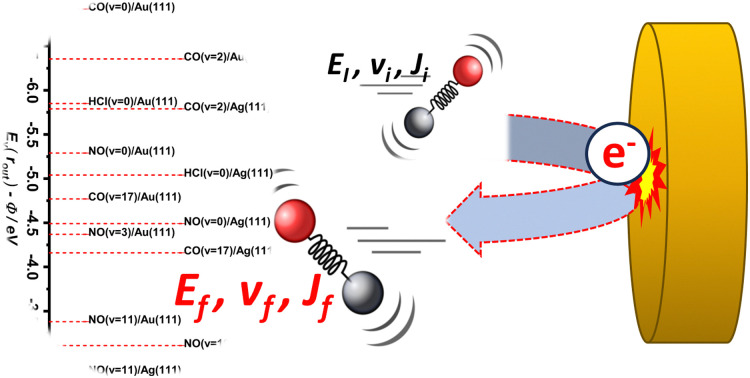

## 1. Introduction

Surface chemistry and heterogeneous catalysis are important and challenging frontiers of modern science, insights from which have vast potential for improvement of industrial processes. One small but important niche in this broad field is the study of vibrational energy transfer between diatomic molecules and metal surfaces. Its importance lies in its role as a model problem for testing theoretical ideas about molecule–surface interactions. The insights gained from the study of vibrational energy transfer in diatomic molecule–metal surface encounters are particularly relevant to understanding the elementary process of bond cleavage, since the vibration of two atoms against one another is precisely the motion needed to induce a reaction.

In fact, experimental observations from this field have brought some of the most fundamental assumptions of theoretical chemistry into question. On insulator surfaces, vibrational energy must be transferred to the substrate's phonons (lattice vibrations), a process that can be well described within the as Born–Oppenheimer approximation^1^ (BOA), the bedrock of computational chemistry.^2^ However, for molecules interacting with metal surfaces, it is now common knowledge that vibrational energy can be much more efficiently dissipated by excitation of electron–hole pairs (EHPs) of the metal.^3,4^ The evidence for this is now overwhelming. It includes observations of Arrhenius surface temperature dependence of vibrational excitation,^5–8^ vibrational excitation of molecules at incidence translational energies (*E*_i_) lower than the vibrational excitation energy,^9^ multi-quantum vibrational relaxation occurring within a fraction of a ps,^10^ and even vibration-induced emission of electrons to the gas-phase.^11–13^ Indeed, the fact that observations of vibrational lifetimes of diatomic molecules on metals are in the 1–2 picosecond time-scale^14–16^ and exhibit Fano lineshapes^17^ and otherwise large linewidths in infrared spectra^18,19^ cannot be explained without invoking excitation of EHPs.^20,21^ In all of these processes, the electronic state of the system changes, meaning that the BOA fails. This begs the question whether any theoretical description based on the BOA can provide an accurate description of the dissociation or formation of a bond in reactions at metal surfaces.

In the early years of this field, a very limited number of observations were available for a very small number of molecules on an equally small number of surfaces. This prevented a systematic understanding as it was not clear that we had explored the entire “phase space of relevant physical parameters”. During the last 10–20 years, this situation has improved dramatically. Indeed, the field has seen a burst of experimental and theoretical studies focusing on vibrationally inelastic scattering of diatomic molecules from metal surfaces. In fact, the currently available quantity of observational information may even strike the student of this field as overwhelming, threatening to shroud the governing physical concepts behind a mask of complexity.

The present manuscript does not pretend to cover the entire wealth of knowledge on BOA breakdown on metal surfaces, which has been reviewed elsewhere.^22–26^ Rather, we hope to convey a unifying view of the mechanisms of vibrational energy transfer at metal surfaces. We accomplish this by focusing on recent experimental and theoretical advances arising from the study of three diatomic molecules: NO, CO and HCl. Exhaustive data for these molecules are now available for both vibrational excitation and relaxation, including experiments with molecules initially excited in low as well as high initial vibrational states. In addition, results are available on two related surfaces: Au(111) and Ag(111) surfaces. The distinct chemical nature of these molecules and metal substrates along with experimental advances led to detailed insight into the dependence of vibrational energy transfer on the nature of the collision partners, molecular orientation, incidence energy and initial vibrational state.

This perspective article is organized as follows. Section 2 briefly surveys the experimental methods now being used that characterize the state-of-the-art. These are based on molecular beams and laser-excitation and detection methods. Section 3 provides a systematic “birds-eye view” of the recent as well as now considered “classic” experimental studies that shaped our understanding of vibrational energy transfer at the molecule/metal interface. This section introduces an intuitive picture that can help us to comprehend and generalize the emerging body of experimental findings in the framework of vibrational energy transfer assisted by transient anion formation. Section 4 sketches briefly the fundamentals of theoretical methods addressing molecule–surface interactions beyond the BOA and highlights recent theoretical advances in vibrational energy transfer of NO, CO and HCl on gold and silver surfaces. Section 5 highlights subtle yet important aspects of translation to vibrational and translation to rotation coupling accompanying electronically nonadiabatic vibrational energy transfer of diatomics at metal surfaces that at present are still not fully captured by the theory. Section 6 provides a brief summary and outlines some future potential experiments that might lead to further understanding addressing open questions.

## 2. Experimental methods

Studies highlighted in this perspective were performed using molecular beams combined with state-of-the-art techniques of laser-based preparation and detection all in combination with surface science methods. The basic concept behind the experiments is to use the incident molecule as a “messenger”. By preparing a molecule with well-defined translational energy and pre-determined vibrational and rotational quantum states, we monitor changes to these quantities resulting from the molecule–surface collision. Analysis of such experiments is analogous to the detective work done at a crime scene – through the knowledge of the state of the system before and after the crime, the process at work during the crime can be discerned. In the remainder of this section, we describe a bit about the tools used in the study of such molecular crime scenes.

### 2.1 Molecular beams and ultrahigh vacuum

Molecular beams have long been a crucial tool in chemical dynamics, as a molecule's speed and direction may be controlled while simultaneously cooling its internal degrees of freedom to a few Kelvin.^27^ The use of molecular beams in problems of dynamics at surfaces has been instrumental in distinguishing direct inelastic scattering from trapping/desorption,^28^ as well as in observations of enhancement of dissociative adsorption probability by increased incidence translational^29^ and vibrational^30^ energy, the influence of molecular alignment on surface reactivity^31^ and Eley–Rideal reactions.^32^

Using beams for problems of dynamics at surfaces requires ultrahigh vacuum (UHV) surface science conditions that are necessary to maintain clean surfaces for long periods of time. The development of beam machines for surface scattering was pioneered in Chicago by Auerbach and Wharton^33^ and developed further by other groups.^34–37^ While this was once an arduous challenge, by using turbomolecular pumps and oil free scroll pumps along with conflat (CF)-flanges to seal chambers together, the construction of such chambers has become so straightforward that one can reasonably argue that it no longer makes sense to build vacuum chambers with O-rings. Without bakeout, base pressures below 10^−8^ mbar are easily reached and with the use of pure materials that can withstand high temperatures, bakeout becomes possible bringing the desired vacuum below 10^−10^ mbar if required. UHV compatible rotatable seals are possible using differentially pumped Teflon rings.^38^ Differentially pumped O-rings can even be used when they are unavoidable as sometimes is the case, for example with optical windows of peculiar shapes.

### 2.2 Preparation of the molecule prior to scattering

Infrared lasers allow excitation of molecules to vibrational states as high as *v* = 3 easily, for example, using Fourier transform limited ns pulsed sources.^39^ To go further up the vibrational ladder, stimulated emission pumping (SEP)^40^ can be used. SEP is an optical double resonance technique, where molecules are excited (pumped) by one laser to an excited electronic state and then “dumped” by stimulated emission using a second laser to vibrationally excited levels in the ground electronic state. In this way, the vibrational quantum number of the molecules can be “dialed in” by appropriately setting the frequency difference between the “pump” and “dump” lasers. Spontaneous emission competes with stimulated emission and may populate undesired vibrational states. This problem can be solved by use of a “sweep” laser that photodissociates the intermediate state used in the SEP a few ns after the pump–dump event is completed.^41^

Highly vibrationally excited CO molecules can be prepared using a scheme referred to as pump–pump–perturb–dump (P3D).^42^ Here, three laser pulses are employed to pump the molecules to a high-*v* state in a ground electronic state. The first two access a high lying level of CO's triplet manifold (X̃^1^Σ → ã^3^Π → ẽ^3^Σ^−^) that is perturbatively coupled to the Ã^1^Π state and the third laser pulse dumps the molecule to the X̃^1^Σ, thus circumventing the need for tunable VUV radiation. The spin-forbidden pump and dump transitions are actually much stronger than might be expected as they both borrow intensity from the very strong Ã^1^Π–X̃^1^Σ transition.

Oriented NO molecules where either the N or O-end points toward the surface can also be produced by combining molecular beam methods with optical excitation. When a high-resolution laser is used to vibrationally excite NO to a single parity state, the molecule will adiabatically orient to any applied electric field lines, which at the metal surface are normal to the surface.^42^ This technique, named optical state selection with adiabatic orientation, is very useful for scattering from metal surfaces.

### 2.3 Detection of scattered molecules

Although laser induced fluorescence^43^ and absorption spectroscopy^44^ have been used with success, probing the final quantum states of scattered molecules is normally performed with resonance enhanced multi-photon ionization (REMPI), which offers higher sensitivity and when combined with TOF mass spectrometry eliminates background from non-resonant ionization. Ion imaging^45^ and state-to-state time-of-flight (TOF)^39^ methods distinguish trapping/desorption from direct scattering, information that is crucially important to the interpretation of the scattering experiments. Beyond this, both methods are capable of providing detailed insights into the intricate interplay between internal and translational degrees of freedom appearing in the scattered molecules. Non-resonant multiphoton ionization (MPI) using high power *f*s lasers is another possible detection method, when state resolved information is not required,^46^*e.g.* for kinetics measurements on surface reactions.^47,48^ Unlike REMPI, MPI has the advantage of being nearly universal.

## 3. Experimental observations

There are many examples of collisions of molecules with metal surfaces where vibrational energy transfer cannot be described within the BOA^1^ and this topic provides the theme of this review. The BOA describes an approximate but indeed fictitious world where electron dynamics is ignored; instead atoms within molecules are envisioned as balls connected by springs. Here, the influence of the electronic structure is bundled into a so-called electronically adiabatic interaction potential, which describes the forces experienced by the nuclei in the average field of the electrons.^2^ In this section we describe many of the observations that characterize electronically nonadiabatic vibrational energy transfer, where the electronic excitation (or de-excitation) of the metal is involved. This type of BOA failure lends itself to experiment and the many observations presented in this section provide a challenge for theories of chemical dynamics at metal surfaces that go beyond the BOA.

### 3.1 The influence of the solid's temperature

In a world of balls and springs, it is impossible for an incident diatomic molecule colliding with a surface to become vibrationally excited unless the kinetic energy from the surface atoms or the molecule's own translational energy is enough to promote the molecule to higher quantum states. One might expect to see an incidence translational energy threshold, below which no excitation to the first excited vibrational state is energetically possible. Indeed, such thresholds have been observed for NH_3_ umbrella vibration excitation occurring in collisions at a Au(111) surface.^49^ In that study, increasing the surface temperature from 300 to 800 K had no influence on the vibrational excitation probability, showing that the coupling of surface atoms’ kinetic energy to molecular vibration is inefficient. This can be understood as due to the frequency mismatch between the phonons of the solid and the molecular vibration – high-order multi-phonon transitions in the solid are required to excite high frequency molecular vibrations.

**Fig. 1:**
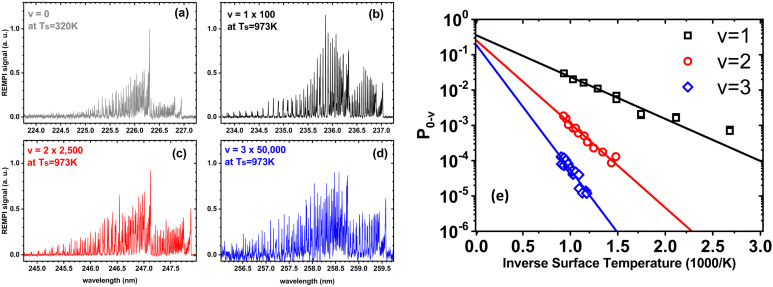
Scattering of NO (*v* = 0) from Au(111) at an incidence energy of translation of 0.4 eV. (a) REMPI spectrum of scattered NO (*v* = 0) at a surface temperature of 320 K; (b)–(d) REMPI spectra of scattered NO (*v* = 1, 2 and 3) when the surface temperature was 973 K. All REMPI spectra are normalized by transition strength, laser power and detection sensitivity. Note the different intensity multiplication factors, indicating different amounts of NO (*v* = 0, 1, 2, 3) stemming from different population of thermally excited electron–hole pairs possessing sufficient energy to excite the corresponding vibrational state; (e) the temperature dependence of the excitation probabilities. Data-points – experiment, solid lines – fits to eqn (1) with *A*_0,1_ = 0.35 ± 0.01, *A*_0,2_ = 0.24 ± 0.01, and *A*_0,3_ = 0.16 ± 0.01. Data replotted from ref. 50.

Fig. 1 shows observations from experiments involving collisions of NO (*v* = 0) with a Au(111) surface at an incidence translational energy of 0.4 eV that produce vibrationally excited states with energies of 0.23 (*v* = 1), 0.46 (*v* = 2) and 0.69 eV (*v* = 3). Note that these are direct scattering events manifested by narrow angular distributions, peaking at the specular angle. The translational energy of incidence is insufficient to explain the observed population of NO in *v* = 2 or 3. Furthermore, mechanical excitation from the solid requires ∼10, ∼20 and ∼30 phonons to produce each of these states, respectively. This is expected to be inefficient.

Unlike NH_3_ collisions with this metal, there is a strong surface temperature dependence of vibrational excitation. The solid lines in Fig. 1 are obtained by fitting the experimentally derived vibrational excitation probabilities to equation1
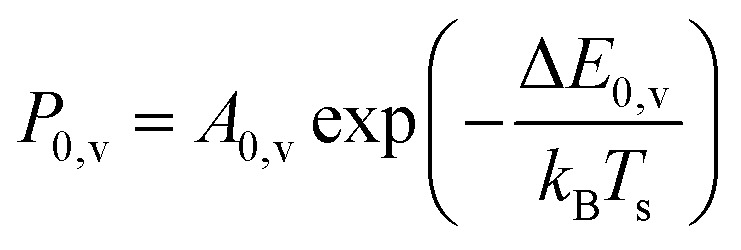
by optimizing *A*_0,v_. Here, *k*_B_ is the Boltzmann constant, *T*_s_ is the surface temperature and Δ*E*_0,v_ is the vibrational excitation energy associated with the collision. Eqn (1) reflects the temperature dependence of the thermal population of excited EHPs,^6,8,9^ which shows that the vibrational excitation of NO on Au(111) involves transfer of energy from excited EHPs to NO vibration and that obedience to eqn (1) is a fingerprint of BOA failure.

### 3.2 The influence of the molecule's incidence energy of translation

**Fig. 2:**
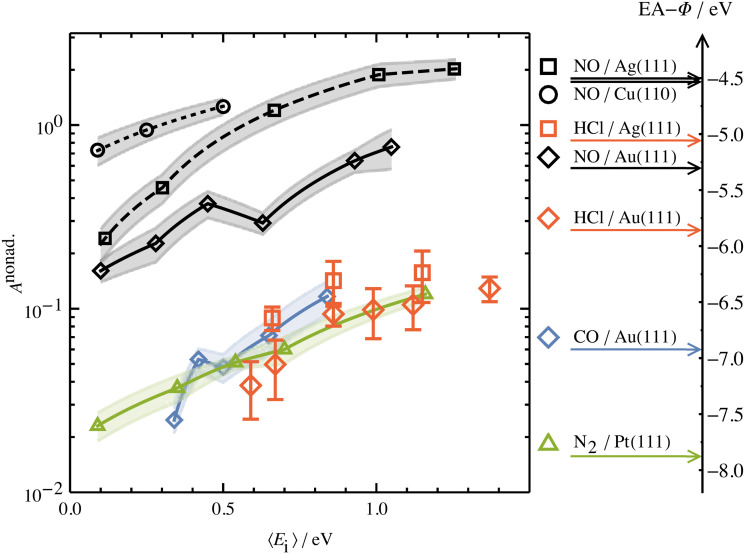
Intrinsic coupling of molecular vibration to metal electrons for seven systems. Here, *A*^nonad^ refers to eqn (2) in the text. From ref. 57 with permission from American Institute of Physics Copyright (2020).

Another experimental observation, which clearly distinguishes between electronically adiabatic and non-adiabatic energy transfer, is the dependence of the vibrational excitation probability on the incidence kinetic energy. Fitting procedures similar to those just described have been applied to seven systems exhibiting electronically nonadiabatic vibrational excitation from *v* = 0 → 1. These seven systems are N_2_ on Pt(111),^51^ CO on Au(111),^52,53^ NO on Ag(111),^8^ Au(111)^54^ and Cu(110)^7^ as well as HCl on Au^55^ and Ag.^56,57^ The resulting values of *A*_0,1_ are shown as a function of the incidence energy of translation for these seven systems in Fig. 2. The incidence energy does enhance vibrational excitation but there is no threshold. Here, the effect of increased incidence translational energy is the increased vibration-EHP coupling strength induced by penetration of the molecule into regions of higher electron density.^5,9^ It is interesting to note that the values of *A*_0,1_ scale approximately with the difference between the molecule's electron binding energy and the solid's work function, a point to which we will return.

**Fig. 3:**
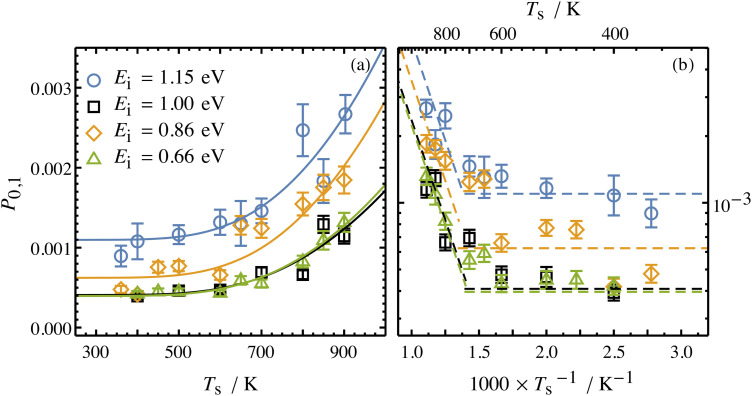
Incidence energy and surface temperature dependence of HCl (*v* = 0 → 1) excitation probabilities for collisions at a Ag(111) surface shown in linear (a) and Arrhenius (b) plots. The nonadiabatic component can be discerned through its strong temperature dependence while the adiabatic component is temperature-independent. From ref. 57 with permission from American Institute of Physics Copyright (2020).

Collisions of HCl on Au(111) and Ag(111) exhibit simultaneous adiabatic and nonadiabatic excitation behavior as has been previously discussed.^55–57^ This is shown explicitly for HCl scattering from Ag(111) in Fig. 3. In this case, the modified version of eqn (1)2
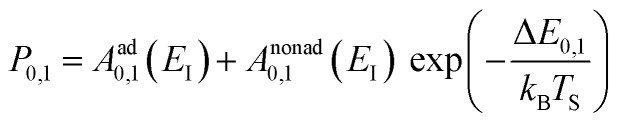
accounting for both effects is used to fit the experimentally derived excitation probabilities, which are shown as the solid lines in Fig. 3.

**Fig. 4:**
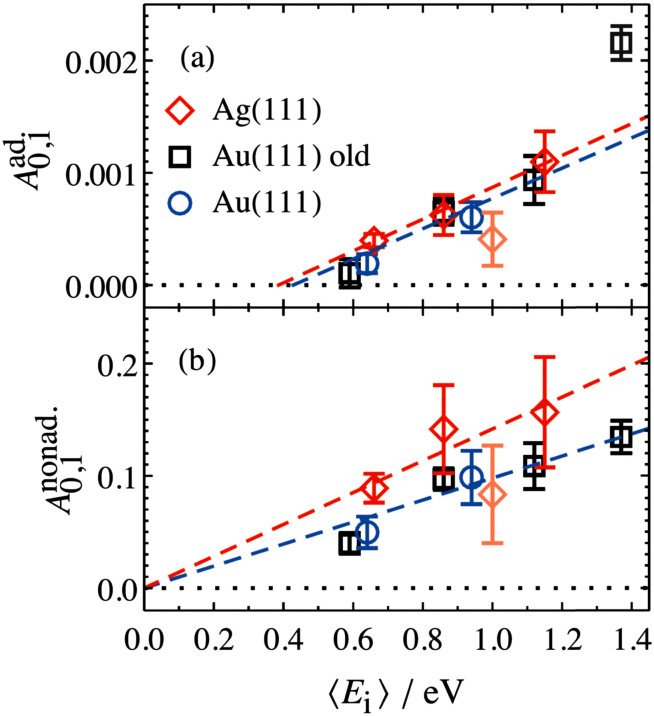
Decomposition of vibrational excitation for HCl interacting with Au and Ag into adiabatic (a) and nonadiabatic (b) components. *A*^nonad^_0,1_ (*E*_I_) extrapolates to zero threshold, while *A*^ad^_0,1_ (*E*_I_) extrapolates to 0.39^+0.05^_−0.08_ eV and 0.43^+0.11^_−0.19_ eV for HCl on Ag(111) and Au(111), respectively. Both thresholds are close to HCl vibrational energy spacing of 0.36 eV. From ref. 57 with permission from American Institute of Physics Copyright (2020).

The values of *A*^ad^_0,1_ (*E*_I_) and *A*^nonad^_0,1_ (*E*_I_) obtained from fitting the HCl vibrational data to eqn (2) help to quantify the importance of nonadiabaticity in energy transfer. See Fig. 4.

The presence of both electronically adiabatic and nonadiabatic excitation mechanisms suggests that a conventional elbow PES is present in this system which mechanically couples *T*–*V* and that, on top of this, transfer of EHP excitation to HCl is also possible. This is consistent with the observations that both adiabatic and nonadiabatic couplings were strongly enhanced by increasing the incidence energy of vibration.^56^ Note that translational energy dependence of the vibrational excitation component stemming from adiabatic coupling exhibits thresholds close to vibrational energy spacing (Fig. 4a). Electronically nonadiabatic vibrational excitation dependence on incident translational energy extrapolates to zero thresholds (Fig. 4b). We note in passing that the mechanism of vibrational excitation of molecules upon collision with metal surfaces was debated in early literature and models, involving reduced dimensionality PESs with judiciously chosen topology parameters, capturing some of the dynamical features of vibrational excitation found in pioneering NO (*v* = 0 → 1)/Ag(111) experiments by the IBM Almaden group,^8,9^ without invoking hot EHPs, were suggested.^58^ However, later experiments on vibrational excitation/relaxation for different molecule/metal surface systems (including multi-quantum vibrational energy transfer and its dependence on molecular orientation),^39,50–54,59–67^ as well as direct observation of large amplitude vibration conversion to electron excitation at metal surface,^11–13,68^ provided undeniable evidence for the importance of electronically nonadiabatic effects. Nevertheless, recent experimental and theoretical works^62,69^ indicate that accurate adiabatic PESs are crucial for quantitative account of the dynamic features observed in vibrational energy transfer and for evaluation of the relative contribution of electronically adiabatic and non-adiabatic channels (see Section 4 for further details).

### 3.3 The influence of incidence energy of vibration

Electronically nonadiabatic vibrational excitation has been the focus of the paper so far, but many experiments have also been conducted with vibrationally excited molecules to observe electronically nonadiabatic vibrational relaxation. These studies profit from the use of overtone pumping and SEP (Section 2.2), which allow initial vibrational excitation to be varied over a large range. An excellent example of this type of study used overtone pumping to produce NO (*v* = 3); these molecules were then scattered from Au(111) to produce *v* = 3, 2 and 1. Observations included vibrationally inelastic (*v* → *v*′) channel – specific scattering angular distributions and translational incidence energy-dependent final vibrational state population distributions,^62^ which are described in more detail later in this section and Section 4, respectively (see Fig. 7 and 16). The angular distributions followed a cos^9^ *ϑ*-form and the most probable scattering angle was not at the surface normal, but rather near the specular angle. This clearly showed that trapping/desorption was unimportant for 0.1 eV ≤ *E*_i_ ≤ 1.2 eV. Similar to electronically nonadiabatic vibrational excitation, this work showed that vibrational relaxation is enhanced by increasing the incidence energy of translation.

**Fig. 5:**
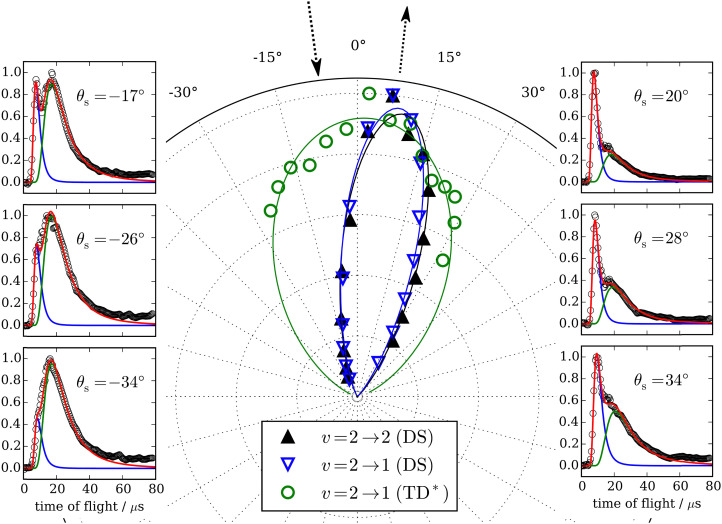
Trapping and thermal desorption of vibrationally excited CO molecules. The panels on the left and right show state-to-state TOF data for the CO (*v* = 2 → *v*′ = 1) channel at indicated scattering angles. The bimodal character is decomposed into two components, the integrals of which are plotted *vs.* scattering angle in the middle panel. The fast component (▲) exhibits a narrow angular distribution and is attributed to direct scattering. The slow component (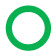) is broad and peaks at the surface normal, indicating trapping/desorption. The incidence energy of translation was 0.32 eV. From ref. 70 with the permission of Springer Nature Copyright (2018).

Similar experiments were performed for CO (*v* = 2 → *v*′ = 2, 1) scattering from Au(111),^70^ and clear fingerprints of electronically nonadiabatic relaxation were seen in a direct scattering mechanism. Here, the incidence translational energy dependence of the relaxation probability is more complex, as trapping takes place at *E*_i_ < 0.4 eV.^53^ Remarkably, vibrationally excited CO molecules were observed to survive trapping followed by thermal desorption^70^ – see Fig. 5 – suggesting that their vibrational relaxation lifetimes were on the order of 10^−10^ s. The lifetime of CO (*v* = 1) on Au(111) was later measured with ultrafast laser pump–probe methods and found to be 49 ± 3 ps,^71^ more than 20× longer than previous observations of CO adsorbed on Cu and Pt^14–16^ and a consequence of the weaker physisorption interaction of CO on Au(111) compared to the chemisorption systems that had been previously reported. A theoretical study showed that the longer lifetime was expected for a physisorbed molecule.^72^

**Fig. 6:**
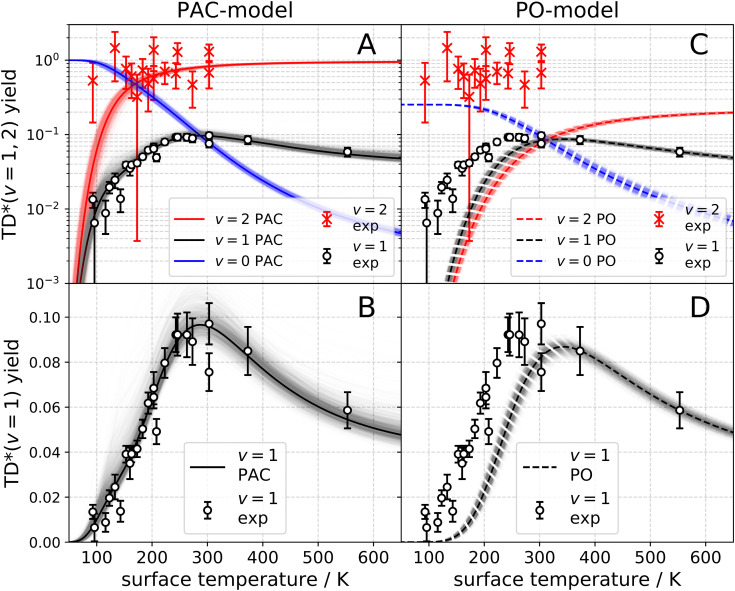
Vibrational state-specific yields of desorbing molecules. (A)–(D) Experimentally observed CO (*v* = 1) (open circles) and CO (*v* = 2) (× symbols) passing through the TD* channel. The error bars indicate a 90% confidence interval. The black and red solid lines are the results of fits to the PAC model. (A) Logarithmic scale. (B) Linear scale. The black and red dashed lines represent the PO model. (C) Logarithmic scale. (D) Linear scale. The blue solid line (PAC) and the blue dashed line (PO) represent the desorbing yield of CO (*v* = 0) stemming from the ultimate vibrational relaxation of CO (*v* = 2). The shading represents the uncertainty of the fit.^74^ From ref. 74 with permission from American Association for the Advancement of Science (AAAS) Copyright (2020).

The observation of trapping-desorption of vibrationally excited CO on Au(111) (TD* for short) showed that the molecules spent sufficient time to equilibrate translationally without equilibrating vibrationally and that the longer vibrational relaxation lifetime of the physisorbed molecule made desorption of vibrationally excited molecules possible. Variation of the surface temperature in these experiments provided control over the surface residence time and hence the probability of the TD* channel. But the CO (*v* = 2 and 1) desorption yield dependence on surface temperature could not be explained by a mechanism involving only a CO physisorption state. See Fig. 6(A) and (B), which show attempts to fit the data with a physisorption only (PO) model. Implications of an adiabatic machine learned PES suggested a way forward – this PES showed that trapping to a physisorption well could proceed in competition with trapping to a chemisorption well at higher energy.^73^ When the chemisorption state was included in the relaxation model, explaining the experimental observations became possible – see Fig. 6(C) and (D) which show the fitting to a physisorption and chemisorption (PAC) model.

This analysis gave a detailed but still simple description of the trapping/desorption mechanism. Under the conditions of the scattering experiments at *E*_i_ = 0.32 eV, all sticking of CO (*v* = 2) is initially into the chemisorption well. At low temperatures (*e.g. T*_S_ = 20 K) the molecule relaxes to *v* = 0 within the chemisorption well, but at higher temperatures 
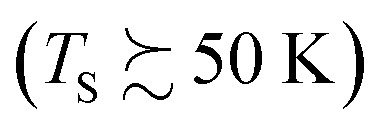
, transfer from the chemisorption to the physisorption well takes place in competition with vibrational relaxation and desorption out of the physisorption well becomes important. By 150 K and higher, the TD* channel happens exclusively by this pathway. It was also possible to perform thermal averaging to obtain thermal adsorption coefficients. An important point is that a large fraction of thermal adsorption proceeds *via* the chemisorption well at all temperatures.

**Fig. 7:**
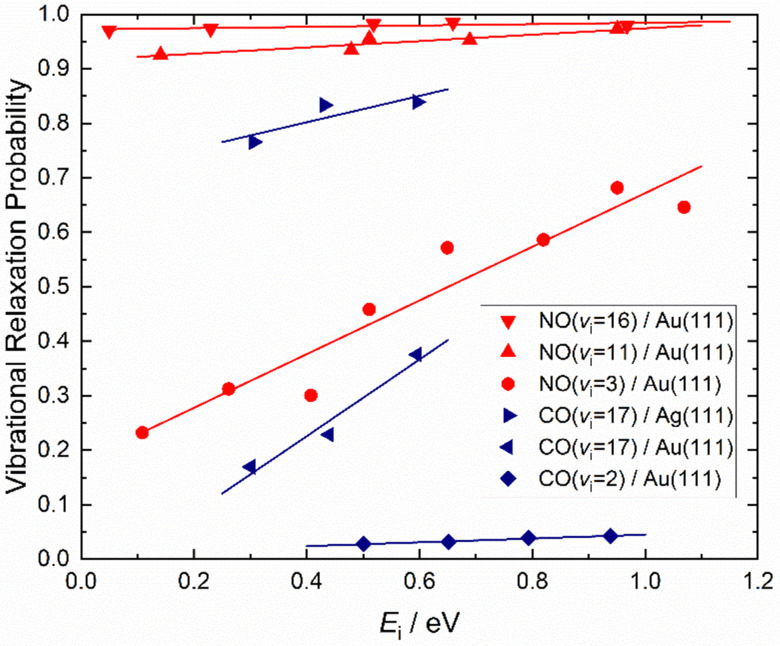
Dependence of the vibrational relaxation of NO and CO on Au(111) and Ag(111) on translational and vibrational energy of incidence (see ref. 63–65 and 76). The work reported in ref. 65 also showed that when NO was initially prepared in *v*_i_ = 16, the vibrational population distribution resulting from relaxation was nearly independent of incidence translational energy – see Fig. 8.

How vibrational relaxation depends on incidence energy of vibration has also been studied for NO and CO. As early as 1990 using SEP, NO was produced in vibrational states 8 < *v* < 25^75^ and vibrational relaxation of NO (*v* = 15) was examined at a single incidence energy of translation in 2000.^10^ With the advent of Fourier transform-limited, high power, frequency stabilized lasers enabling efficient preparation of low vibrational states by overtone pumping, and development of the pump–dump–sweep^41^ and pump–pump–perturb–dump (P3D)^42^ methods enabling access to high vibrational states of NO and CO, systematic studies could be made. Fig. 7 shows one of the important outcomes of this work; the dependence of vibrational relaxation on translational incidence energy becomes substantially weaker at high vibrational incidence energy.^65^

**Fig. 8:**
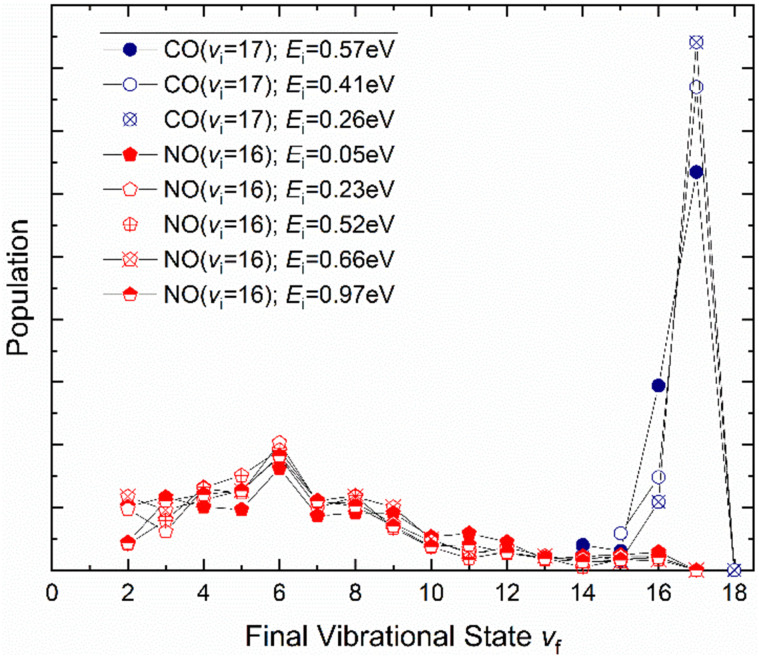
Vibrational state distributions resulting from the scattering of NO (*v* = 16)^65^ and CO (*v* = 17)^63^ from Au(111) for several incidence translational energies.

It is interesting to contrast this with the trends seen for CO (*v*_i_ = 17) molecules colliding with a Au(111) surface. See Fig. 8. Overall, the relaxation probability is smaller than that of NO as is the amount of energy transferred. Similar to NO (*v* = 3) on Au(111), the survival probability of CO (*v* = 17) on Au(111) depends on incidence translational energies and so does the vibrational population distribution.^63^

### 3.4 Intermezzo: the electron transfer picture

**Fig. 9:**
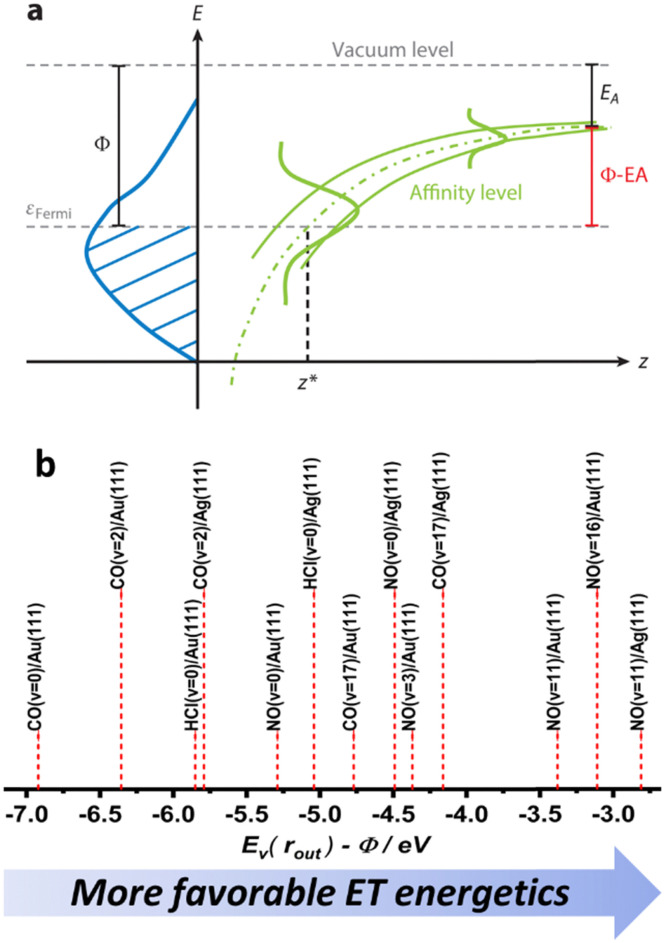
(a) The electron transfer (ET) picture. From ref. 23 with permission from Annual Reviews Copyright (2015). (b) The energetics of ET for some of the molecule surface systems highlighted in this work, ordered according to *E*_v_(*r*_out_) − *Φ* difference – as a qualitative proxy for EHP-V coupling strength (see ref. 57 and 64).

Despite their similarities, the observations made for NO (*v* = 16) and CO (*v* = 17) vibrational relaxation exhibit striking differences. These differences can be rationalized within a mechanism of vibrational energy transfer that involves electron transfer (ET).^10,11^ As the molecule approaches the surface, it reaches a critical distance z* at which its affinity level (defined as the difference between the vertical binding energy at the outer turning point and metal's work function, *E*_v_(*r*_out_) − *Φ*) is compensated by image charge stabilization (ICS) and crosses the Fermi level of the metal – see Fig. 9. Here it may capture an electron from the solid.

The resulting transient anion continues to vibrate along a diatomic potential that is obviously much different from that experienced by the neutral molecule. The ET event induces a force between the atoms that continues until the electron is transferred back to the solid. In this picture, the vibrational relaxation probability is higher for NO than for CO as the ET event is more likely for NO than for CO. This reflects NO's higher electron binding energy, which allows it to capture an electron at a distance on its approach to the solid that is further away than for CO. The much larger number of quanta transferred in the case of NO compared to CO results from the longer lifetime of the transient NO^−^ compared to its CO^−^ counterpart. Here the argument is qualitatively the following. If CO^−^ were to live only a small fraction of the vibrational period, whereas the transient NO^−^ might live say 1/2 the vibrational period, then the time-integrated force (impulse) exerted on the bond is much reduced for CO compared to NO. This leads to a much smaller change in relative momentum between the two atoms. This simple classical picture is consistent with what we know about the two anions – NO^−^ is stable while CO^−^ is in an unbound resonant state. It is also noteworthy that the vibrational relaxation exhibits a lack of incidence translational energy dependence in the NO case, whereas for CO, increasing the incidence energy enhances vibrational relaxation. This can also be explained by an ET picture, where ET occurs for NO at a long distance from the surface, which is reminiscent of a harpooning reaction, whereas the CO molecule must travel much closer to the surface and partway up the repulsive wall of the interaction potential with the surface before ET is possible. In this picture it is also plausible that the CO^−^ is shorter lived.

This picture of electronically nonadiabatic vibrational energy transfer is highly simplified but qualitatively correct. There are additional subtleties that arise when a proper quantum picture is developed, but it is important that the reader keep this picture in mind when considering the rest of this review and this field – it can take you far.

### 3.5 The influence of the solid's work function

An obvious test of the ET picture of electronically nonadiabatic vibrational energy transfer would be to vary the solid's work function. Here, the assumption is that the lower the work function of the solid, the higher the probability for ET.

**Fig. 10:**
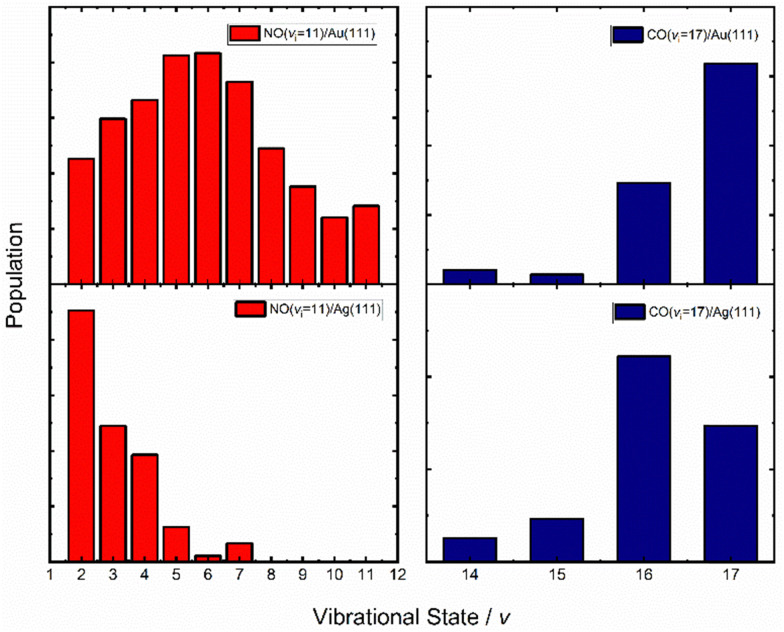
Vibrational state distributions of NO (*v* = 11, *E*_i_ = 0.51 eV) (left) and CO (*v* = 17, *E*_i_ = 0.57 eV) (right) scattered from two solids with different work functions – Au(111) (top) and Ag(111) (bottom). The vibrational state distribution is drastically shifted toward lower vibrational states in the case of Ag(111). Data replotted from ref. 64 and 67.

Fig. 10 (left) shows vibrational distributions produced when NO (*v* = 11) collides with either Au(111) (top left) or Ag(111) (bottom left), respectively.^67^ The work function of Au(111) is 5.33 eV while that of Ag(111) is 4.53 eV.^77^ The effect is striking. Silver, with its lower work function and lower barrier for NO dissociation,^78^ results in a vibrational distribution where the initial state is now undetectable; furthermore, due to background effects, it cannot be ruled out that the most highly populated final vibrational state is *v* = 0. State-to-state TOF measurements were carried out on the NO (*v* = 11 → 4, 3 & 2) scattering channels. The vibrational energy release in these three cases was 1.5, 1.7 and 1.9 eV, respectively; nevertheless, the average translational energy of the scattered molecules was only 0.5 eV, remarkably close to the incidence energy of translation, 0.51 eV. This is an unmistakable sign of significant vibrational energy transfer to the solid, creating excited EHPs, consistent with the ET picture.

While in the early work^10^ the similarity of the average translational energy of the vibrationally inelastically scattered NO to the incidence energy of translation was interpreted as the spectator character of translation DOF, our current understanding is that this is only approximately true. For example, nearly unaltered translational energy (∼0.5 eV) seen for NO (*v* = 11 → 4, 3 & 2)/Ag(111) scattering^67^ results from compensation of translational inelasticity (T-phonon energy transfer, that amounts to ∼0.7 × *E*_i_, according to the Baule limit expectation^61^) by partial *V*–*T* energy transfer – (0.17 ± 0.1)Δ*E*_vib_ – appearing as outgoing NO translation.^67^ Furthermore, translational energies as high as 1 eV were seen in the scattered NO (*v* = 11 → 4, 3 & 2) molecules translational energy distributions, a clear sign of *V*–*T* energy transfer.^67^ In addition, direct observation of *T*–*V* and *V*–*T* energy transfer was also seen in vibrationally inelastic collisions of NO (*v* = 2, 3) with Au(111);^39^ the mechanism behind this coupling remains unclear. We shall return later to this subtle yet important topic of *V*–*T*/*T*–*V* and *T*–*R* transfer accompanying V-EHP coupling – see Section 5.

A similar comparison could be made for the vibrational relaxation of CO (*v*_i_ = 17) in collisions with Au(111) and Ag(111)^64^ and the results are qualitatively similar to those seen for NO colliding with Au(111) and Ag(111) – see Fig. 7 and 10 (right). Specifically, the survival probability of CO (*v*_i_ = 17) is ∼4× larger for collisions on Au compared to Ag and for the latter, a larger average number of quanta are transferred to the solid than for the former.

The dependence of ET-driven V-EHP coupling on the solid's work function is also seen in vibrational excitation, as discussed in Section 3.2 in the context of the influence of incident translational energy. Compare, for instance, the pre-exponential factors *A*_01_ of the Arrhenius expressions for vibrational excitation of NO (*v* = 0 → 1) on Au(111) *vs.* Ag(111) (see Fig. 2) and HCl (*v* = 0 → 1) on Au(111) *vs.* Ag(111) (see Fig. 2 and 4b).

Even clearer evidence of the influence of the solid's work function was found when using samples of atomically thin Ag films grown on Au(111). Due to the demonstrated layer-by-layer growth of Ag on Au,^79–81^ samples can be prepared with a defined number of Ag atomic layers, allowing control of work function.^80,82^ The scattering of NO (*v* = 2) (prepared by overtone pumping) from atomically defined thin silver films grown on Au(111) was reported in 2018.^83^ The translational incidence energy of NO was chosen to be 0.59 eV to avoid trapping-desorption. Direct scattering was confirmed from narrow scattering angular distributions. Controlling the thickness of the films provides a way to systematically vary the work function between that of Au and Ag. In these experiments after scattering from a surface at room temperature, the initially prepared NO (*v*_i_ = 2) remains in the same (*v* = 2 → 2) or relaxes into lower vibrational states (*v* = 2 → 1, 0). Quantitative information on the probabilities for vibrational survival P (*v* = 2 → 2) and relaxation P (*v* = 2 → 1, 0) was obtained from the acquired state-selective REMPI signal strengths.

**Fig. 11:**
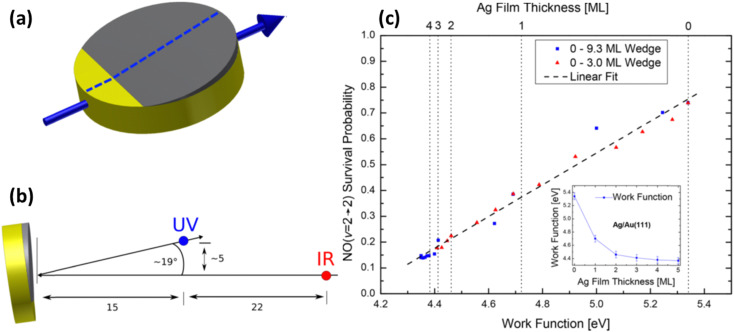
Measurement of vibrational survival probability *vs.* systematically varied work function of the solid. (a) Wedged (not to scale) Ag thin film on Au(111). Film thickness is increasing when moving along the surface in the direction of the blue arrow. Scattering experiments have been performed along the center of the crystal (dashed line) enabling probing the locations with different work functions by the incident NO (*v* = 2) molecular beam. (b) Positions of the UV REMPI (blue) and the IR-overtone pumping (red) beams relative to the surface and the molecular beam (represented by single-headed black arrows), used for quantification of vibrational survival probability. The laser beams are normal to the molecular beam and the surface normal. (c) Survival probability of NO (*v* = 2) scattered from Ag/Au(111) plotted against the surface work function (see the inset for work function correlation with Ag film thickness). From ref. 83 with permission from American Chemical Society Copyright (2018).

Specifically, it was observed that as the Ag layer thickness increased from 0 to 3 atomic layers, the survival probability of NO (*v* = 2) decreased by one order of magnitude – see Fig. 11. Beyond 3 ML of Ag, the values remained unchanged, which was interpreted as having reached the bulk limit. Remarkably, kinks in the vibrational survival probability were seen as the 1st and 2nd atomic layers closed. A simple surface induced dipole model of layer-by-layer growth predicts similar kinks in the work function.

**Fig. 12:**
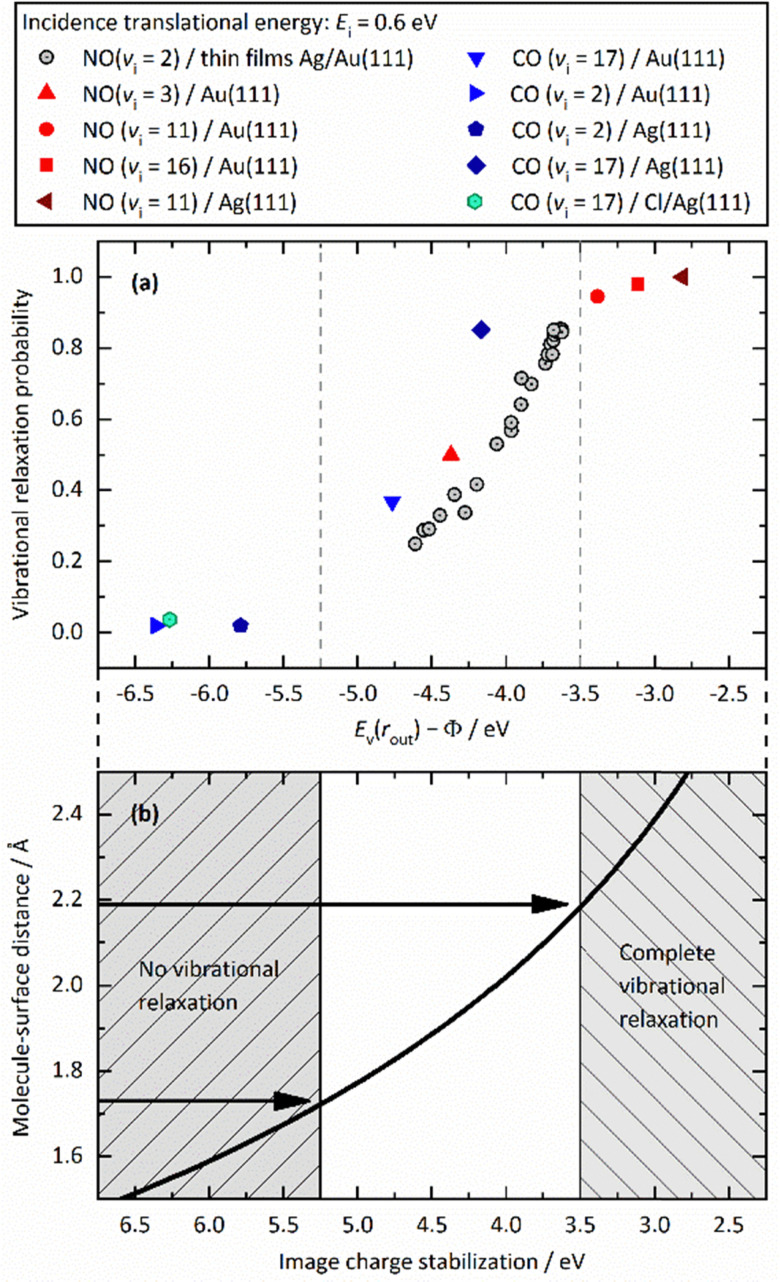
(a) Relaxation probability of various molecule–surface systems as a function of *E*_v_ − *Φ* at an incidence translational energy of 0.6 eV. The results are shown for CO (*v*_i_ = 17)/Ag(111), CO (*v*_i_ = 17)/Au(111), CO (*v*_i_ = 2)/Ag(111), CO (*v*_i_ = 2)/Au(111), NO (*v*_i_ = 11)/Ag(111), NO (*v*_i_ = 3, 11, 16)/Au(111), and NO (*v*_i_ = 2) on thin films of Ag on Au(111). All systems follow a common trend showing an increase of the relaxation probability from 0 to 1 between −5.25 eV and −3.5 eV (b) Image charge stabilization (ICS) of a negatively charged molecule in the vicinity of the surface. The ICS must compensate for the difference *E*_v_(*r*_out_) − *Φ* to make the ET energetically feasible. Little vibrational relaxation occurs for ICS values below −5.25 eV as the molecule cannot get closer to the surface and approaches the repulsive wall at which it is scattered back. For values above −3.5 eV, the molecule remains long enough in regions of electronic non-adiabaticity so that complete vibrational relaxation occurs. The arrows indicate corresponding molecule–surface distances. Reproduced from ref. 64 with permission from the PCCP Owner Societies.

Fig. 12 unifies the results obtained from different vibrational relaxation experiments performed over the recent decade. Vibrational relaxation probabilities are shown *versus* the asymptotic electron affinity level of the molecule at its outer classical turning point of vibration, *E*_v_(*r*_out_) − *Φ*. *E*_v_(*r*_out_) varies from molecule to molecule (here NO and CO) and according to its initial vibrational state (2 ≤ *v*_i_ ≤ 17). Along with the work function *Φ*, which can be varied by the choice of the substrate (Au, Ag and Au with varying Ag coverage), the energetics of electron transfer can be controlled. Since the vibrational relaxation probability depends on the incidence energy of translation, all values are shown for *E*_I_ ∼ 0.6 eV, to compare different scattering systems on an equal footing.

We observe an S-shaped behavior that describes a correlation across all of the studied systems. When the difference of vertical binding energy and work function is small (strongly negative values on the left hand side of panels a and b), electron transfer from the metal to the molecule is unfavorable and little relaxation occurs. In contrast, for low work function values and high electron vertical binding energies, the difference is large, and ET and the resulting vibrational relaxation are facilitated. Recall that the ICS must compensate the value of *E*_v_(*r*_out_) − *Φ* to make the ET energetically feasible. Values below −5.25 eV cannot be achieved by ICS as the molecule cannot get closer to the surface and approaches the repulsive wall at which point it is scattered back leading to no or little vibrational relaxation. For values above −3.5 eV, the molecule remains long enough in regions of electronic non-adiabaticity so that complete vibrational relaxation occurs. The arrows indicate corresponding molecule–surface distances.

The lowest work function material from which vibrationally inelastic scattering has been observed is a Cs dosed Au(111) surface, with a work function of 1.61 ± 0.08 eV^84^ (not shown in Fig. 12). When SEP was used, vibrational relaxation of NO (*v*_i_ = 22 and 16) resulted in electron emission.^11,12^ Using a retarding energy analyzer, the electron energy distributions could be determined.^68^ This showed that multi-quantum vibrational relaxation (10 < Δ*v* < 18) was responsible for electron emission.

In particular, the observations using atomically controlled thin silver films and the vibrationally promoted electron emission results are some of the strongest evidence for an ET mechanism in the electronically nonadiabatic vibrationally inelastic scattering of molecules from metals. We emphasize that one electron accepts all of the vibrational energy being transferred.

### 3.6 Inverse velocity dependence of electron emission

Interestingly, the quantum yield of vibrationally promoted electron emission from the Cs-dosed Au(111) surface was found to have an inverse dependence on the velocity of the incident vibrationally excited NO molecules.^13^ This may appear to contradict previous observations, where both vibrational relaxation and excitation probabilities were found to grow with incident energy of translation (see Sections 3.2 and 3.3). This apparent contradiction is however easily understood in terms of a “window of opportunity mechanism” and is a result of the fact that exo-electron emission occurs only when an electron is produced with enough energy to escape the surface, while vibrational relaxation can be observed even when the autodetached electron does not escape the surface. Due to the low work function of Cs/Au, ET to NO (*v* = 18, *r*_out_) becomes possible already at a distance from the surface of *z* ∼ 10 Å.^13^ As the anion's bond recompresses, the auto-detachment of the electron results as it accepts vibrational energy from the molecule. Due to the increasing image charge attraction upon approach to the surface, the electron may only escape to the vacuum level for *z*_c_ > 4.8 Å.^13^ Hence the time the anion spends between *z* ∼ 10 Å and 4.8 Å, which is inversely proportional to its velocity of incidence, determines the probability for electron emission.

### 3.7 The influence of molecular orientation

If ET is the fundamental mechanism for electronically nonadiabatic vibrational energy transfer, steric effects are to be expected.^85^ These arise due to the spatial distribution of the lowest unoccupied molecular orbital (LUMO), which although delocalized is found preferentially on the N-atom end of the molecule. This is also the reason why NO binds to Au with the N-atom closest to the surface.^85^

**Fig. 13:**
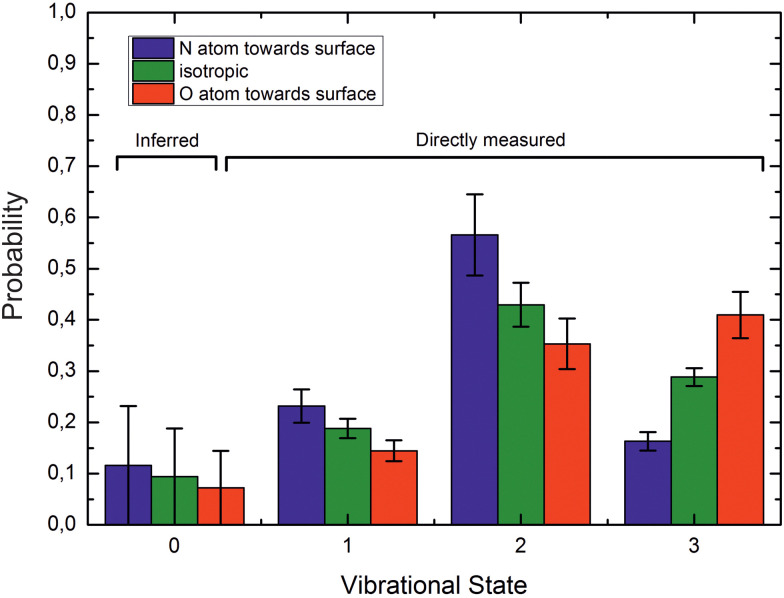
Vibrational state distributions after scattering oriented NO (*v* = 3) from Au(111). Vibrational relaxation is significantly enhanced when scattering N-atom first. *E*_i_ ∼ 0.9 eV. Reproduced from ref. 59.

Experimental studies using molecular beams of oriented NO molecules were made possible by optical state selection with adiabatic orientation.^42^Fig. 13 shows scattering results for oriented NO (*v* = 3) from Au(111). When the N atom is oriented toward the surface, vibrational relaxation is favored; on the other hand, when the O atom is oriented toward the surface, vibrational relaxation is suppressed. The scattering vibrational distribution for randomly oriented NO is also shown for reference. The high incidence energy prevents reorientation of the dipolar molecule upon its approach to the surface.

**Fig. 14:**
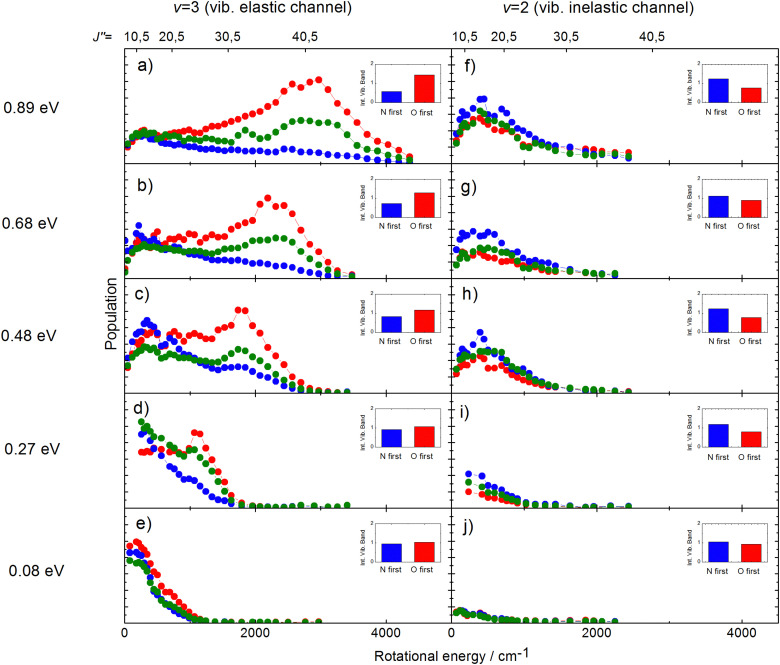
Rotational state distributions for NO (*v* = 2) and NO (*v* = 3) for five different translational energies after scattering NO (*v* = 3) from a Au(111) surface. The different colors denote N-first (blue), isotropic (green), and O-first (red) collisions. The rotational rainbow observed in the vibrationally elastic channel does not appear in the vibrationally inelastic channel. The insets show the integrated band intensities for both orientations and hence reflect the *v* = 3 vibrational state survival probability (panels a,b,c,d,e) and the vibrational relaxation probability to NO (*v* = 2) (panels f,g,h,i,j), respectively. The band intensities in the insets are scaled relative to the integrated band intensity of the isotropic signal. From ref. 60 with permission from American Institute of Physics Copyright (2014).

The rotational distributions of the scattered molecules were also seen to depend strongly on orientation and the orientation dependent rotational distributions were strongly dependent on incidence energy of translation.^60^ See Fig. 14, showing the rotational distributions for NO (*v* = 3 → 3, 2)/Au(111) scattering channels.

The potential energy surface (PES) of NO on Au(111) exhibits a repulsive interaction when the O-atom is oriented toward the surface, whereas it is attractive when the N-atom is closest to the surface.^69^ Consequently, when the O-atom points toward the surface in the collision, peaks in the distributions at high rotational quantum number, referred to as rotational rainbows, are seen. Remarkably, rotational rainbows are only seen for vibrationally elastic channels. This makes clear that vibrational relaxation of NO (*v* = 3) occurs predominantly when the N-atom points toward the surface, consistent with the results shown in Fig. 13. One also sees that the rainbows disappear at reduced translational energies of incidence. This coincides with the disappearance of the influence of initial orientation on the vibrational relaxation probabilities and rotational distributions. This is a clear sign of dynamical steering, where the torque experienced by the NO molecule due to interaction with the surface is strong enough to reorient it during the collision. This torque is obviously quite small as even an incidence energy of translation at or above 0.3 eV is enough to suppress the steering.

**Fig. 15:**
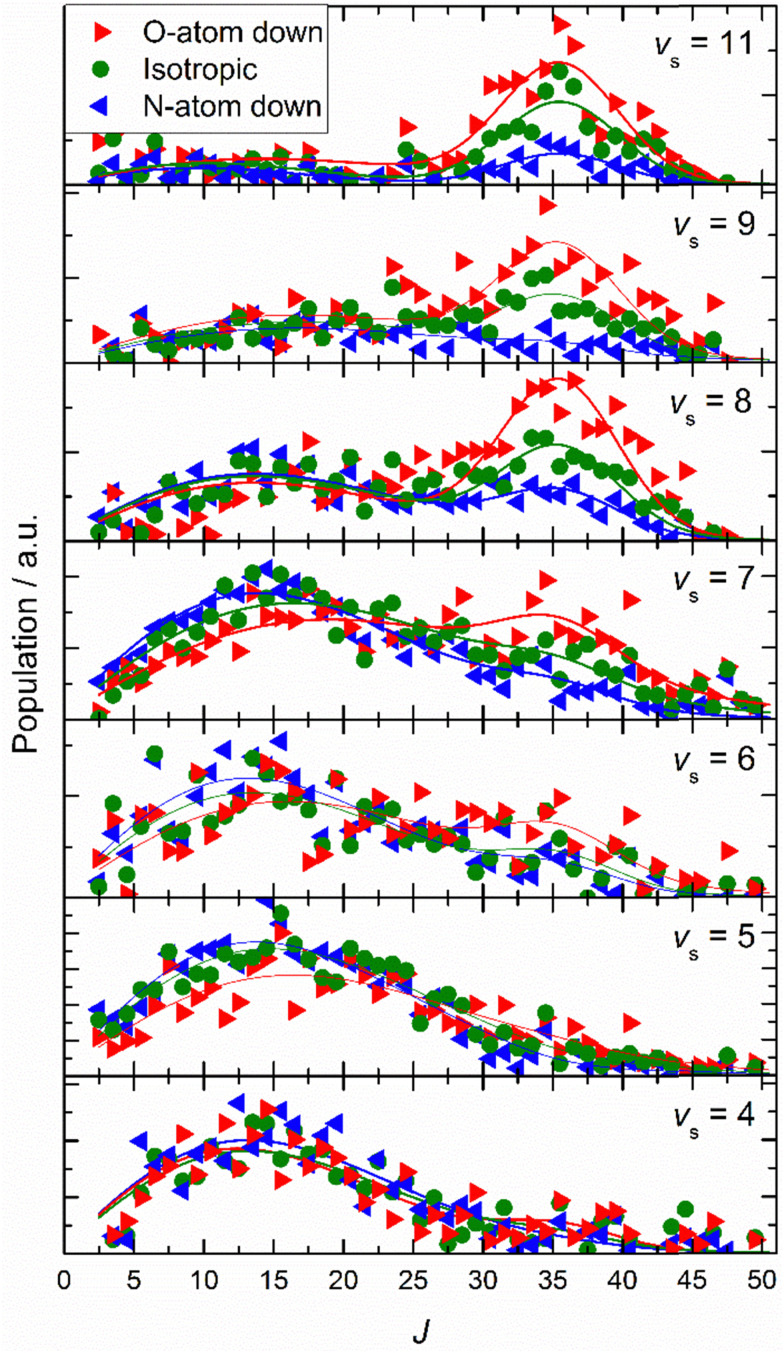
Final rotational state population distributions are influenced by incidence orientation. NO (*v*_i_ = 11, *J*_i_ = 0.5) approaches the surface with 0.51 eV incidence translational energy. Rotational state distributions are shown for scattered NO molecules into several vibrational states. Three orientation cases are shown: N-first (blue), O-first (red) and unoriented (green). The peaks near *J* ∼ 35 reflect rotational rainbows arising from collisions where the O-atom points toward the gold surface. Solid lines are drawn to guide the reader's eye. Reproduced from ref. 66 with permission from the PCCP Owner Societies.

Similar experiments were carried out for oriented NO (*v*_i_ = 11) at a translational incidence energy of 0.51 eV^66^ – see Fig. 15. In contrast to the work with NO (*v*_i_ = 3), here rotational rainbows are also seen for vibrationally inelastic scattering.^66^ This shows that ET is possible for NO (*v*_i_ = 11) even when the O atom is pointed toward the surface, something that is impossible for NO (*v*_i_ = 3). This is fully consistent with the ET picture described above, as increasing vibrational excitation leads to higher vertical electron binding energy for the NO molecule at its outer classical vibrational turning point. One can also discern from these data that the magnitude of the vibrational energy transferred is greater for NO molecules with their N-atoms pointed toward the surface; presumably such molecules can approach the surface more closely and this leads to a larger average number of quanta being transferred.

The observations described above provide a basis for the development of dynamical theories that no longer rely on the BOA. We next review recent progress made in this direction.

## 4. Theory that goes beyond the Born–Oppenheimer approximation

The current understanding of the energy transfer between atoms or molecules and metal surfaces suggests the active involvement of the solid's EHPs. As early as 1979 theorists speculated that sticking of atoms and molecules to metals could not be understood in the absence of electronic dissipation pathways^86^ and in 1985 seminal experiments on NO scattering from Ag(111)^9^ strongly suggested EHP-mediated vibrational energy transfer. A simple physical picture eventually emerged to explain these experiments, wherein electron transfer to the NO molecule produces a transient negative ion, which when neutralized by an electron transfer back to the metal may find itself in another vibrational state. The vibrational transition arises from the large interatomic force exerted on the N and O atoms during the two charge transfer events.

This picture can be formalized within a Newns–Anderson electronic Hamiltonian:^5,87^3
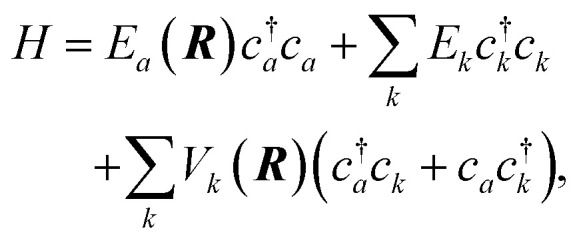
where the affinity level of the incident molecule is described by fermion creation (annihilation), represented by the operator *c*^†^_*a*_ (*c*_*a*_), while the creation (annihilation) operator *c*^†^_*k*_ (*c*_*k*_) refers to excitation/de-excitation of the *k*th metal surface electronic eigenstate. The most important property of the Newns–Anderson Hamiltonian is the dependence of the energy of the affinity level *E*_*a*_(***R***) and the mixing coefficients for molecular and metal electronic states *V*_*k*_(***R***) on the nuclear geometry represented by the vector ***R***. *E*_*a*_(***R***) describes the affinity level stabilization with the approach of the molecule to the surface as well as its dependence on distortions of the molecular structure.

The straightforward solution of the Schrödinger equation for all system's degrees of freedom is a formidable task, which requires further assumptions. One of the most effective approaches considers the nuclear subsystem as producing an external time-dependent field perturbing the electronic degrees of freedom, thereby assuming that the motion of the nuclear degrees of freedom can be described by a classical trajectory ***R***(*t*) moving on a potential energy surface corresponding to the current electronic state of the system. The time-dependent coupling in the Newns–Anderson Hamiltonian then induces electronic transitions, which are modelled with the so-called Tully surface-hopping method.^88^ This provides a powerful and computationally efficient tool that can be implemented with an independent-electron surface hopping (IESH) algorithm.^87^ A crucial ingredient of the IESH method is a set of many-dimensional potential energy surfaces for the electronic states of the system, whose construction, while rather demanding, can be carried out.^87^

If one is concerned with vibrational relaxation lifetimes of adsorbed molecules, it is possible to apply a more drastic approximation, which allows the use of perturbation theory. Here, the vibrational motion of the adsorbate is considered as a small perturbation inducing the coupling to the EHPs of the metal, which allows the use of a linear approximation for describing the dependence of the electronic Hamiltonian (3) on the nuclear degrees of freedom. Invoking Fermi's Golden Rule, we then obtain the vibrational lifetime:4
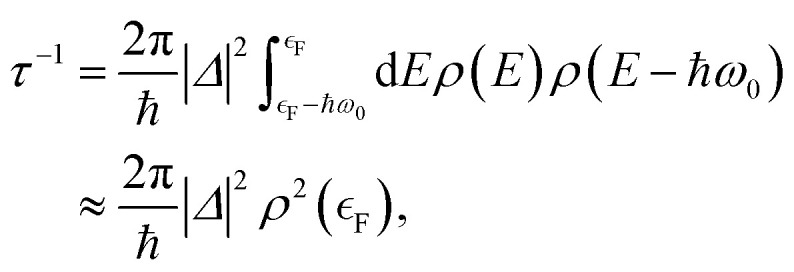
where *Δ* is the vibrational–electronic coupling, *ρ*(*E*) is the density of electronic states projected on the affinity orbital of a molecule, *ω*_0_ is the vibrational frequency, and 
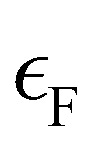
 is the Fermi energy.

Further simplification is possible if we allow only low-energy EHPs to accept energy from the motion of the nuclei.^89^ Then in the Markovian limit, the dynamics of a projectile with mass *m* and position ***r*** is governed by the Langevin equation5
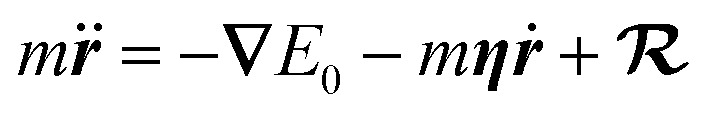
on a properly defined PES *E*_0_, with nonadiabaticity introduced on the level of electronic friction. Here, the matrix of friction coefficients ***η*** depends on the nuclear positions, and the stochastic force 
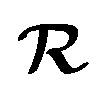
 represents a white-noise spectrum that is directly related to the friction coefficient by the fluctuation–dissipation theorem. Eqn (5) shows that nuclei feel the presence of EHPs as a drag force dissipating their energy into the electronic bath in the presence of a random force that ensures thermal equilibrium may be reached. This approach has the advantage of simplicity as we may ignore the dynamics of electronic degrees of freedom.

Probably, the most common method to get the information on the electronic friction tensor ***η*** entering eqn (5) stems from studies of a proton moving in a metal, which has been modeled as a homogeneous electron gas.^89–91^ Due to the symmetry of this simple system, the friction tensor becomes a single number – a friction coefficient – and is related to the electron transport cross-section at the Fermi surface by means of the energy loss function (stopping power). This leads to the opportunity of determining the friction coefficient as a function of the background electron density, which can be derived from the DFT calculations in the local density approximation.^89,91–93^ Hence, this technique is usually referred to as local density friction approximation (LDFA). Due to its simplicity and computational efficiency the LDFA has become very popular when solving problems that rely on eqn (5). It has been particularly successful in problems of atomic scattering from metals – the translational energy loss spectra calculated in the framework of this approach agree well with the measured ones in the case of *H*(*D*) atom scattering from a series of transition metal surfaces^94–99^ and from the oxygen-covered Pt(111).^100^ See ref. 101 for a review.^101^ The LDFA method was also applied to study the influence of the non-adiabatic effects on reactivity (dissociative chemisorption and recombination) of diatomics at surfaces.^102,103^

A more advanced and straightforward approach to the calculation of the electronic friction was developed by Head-Gordon and Tully.^104^ They derived eqn (5) from a semiclassical approach, where the time-dependent Schrödinger equation with nonadiabatic coupling governs the dynamics of electrons, while the nuclei move classically on an effective adiabatic potential energy surface. They found that in the weak coupling approximation, the friction tensor can be expressed in terms of orbital-dependent nonadiabatic coupling in a fashion resembling Fermi's Golden Rule similar to eqn (4). This approach—often referred to as molecular dynamics with electronic friction with orbital-dependent friction (MDEF-ODF) – is much more computationally demanding than LDFA, as the explicit information on the molecular orbitals is necessary. On the other hand, it allows going beyond the isotropic approximation inherent to LDFA^105–108^ – the detailed analysis of the friction tensor in the case of CO adsorbed on Cu(100) revealed that the damping of C ↔ O vibration promotes energy transfer between the adsorbate and surface vibrational modes, a direct result of the fact that the friction tensor is anisotropic and nondiagonal.^107^

**Fig. 16:**
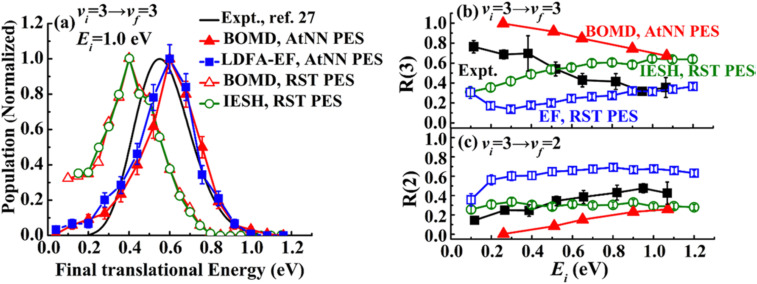
Comparison of recent theoretical calculations with experiment for NO collisions with Au(111). (a) Comparison of experimental final translational energy distributions from ref. 109 (black curve) of NO (*v*_i_ = 3 → *v*_f_ = 3) scattering from Au(111) at *E*_i_ = 1.0 eV and *T*_S_ = 320 K with theoretical results from BOMD (red filled triangles) and LDFA-EF (blue filled squares) using a new NN-PES by Bin Jiang and coworkers^69^ and BOMD (red open triangles) and IESH (green open circles) simulations using original PES that was suggested to be too soft in our previous work.^62,109^ (b) and (c) Experimental branching ratios (black filled squares) of (b) NO (*v*_f_ = 3) and (c) NO (*v*_f_ = 2) scattered from NO (*v*_i_ = 3)^62^ are compared with BOMD and EF ones using NN-PES,^69^ and EF (blue open squares) and IESH (green open circles) results in ref. 62, as a function of *E*_i_ at *T*_S_ = 300 K. The branching ratio is defined as *R*(*v*_f_) = *S*(*v*_f_)/(*S*(*v* = 1) + *S*(*v* = 2) + *S*(*v* = 3)), where *S*(*v*_f_) is the scattering probability to a final vibrational state (*v*_f_). The new NN-PES resulted in nearly perfect agreement with the experiment with respect to translational inelasticity (a) and better prediction of the trend observed for vibrational relaxation probability dependence on the incident translational energy. From ref. 69 with permission from American Chemical Society Copyright (2019).

The IESH approach has been applied to describe dynamical steering and vibrational energy relaxation of highly vibrationally excited NO molecules scattered from Au(111)^85^ as well as to the multi-quantum vibrational excitation of NO molecules with incidence energies from 0.1 to 1 eV scattered from the Au(111) over a wide range of surface temperatures.^54^ Progress in the detailed understanding of the nonadiabatic energy transfer between the molecular vibrations and the metal's EHPs motivated experiments with improved accuracy in measuring the vibrational (de-)excitation probabilities’ dependencies on molecular translational and rotational degrees of freedom.^39,61,62^ The experiments revealed clear deficiencies in the original implementation of IESH.^85^ Detailed analysis of IESH trajectories suggested that the adiabatic PESs used in the original approach were too corrugated or soft. This led to predictions of multi-bounce collisions, for which there is no evidence.^109^ The theoretical results were not in agreement with experimentally observed angular and translational energy distributions of scattered NO molecules from the Au(111) surface. Moreover, they even failed to reproduce final kinetic energy distributions for simple adiabatic vibrationally elastic scattering – see Fig. 16.

This prompted several theoretical groups to construct more accurate adiabatic and diabatic PESs paying close attention to the choice of the DFT functional and using neural network methods to reduce the fitting error. Several theory groups produced a set of such potentials with impressive accuracy for NO at Au(111),^69,110–113^ NO at Ag(111),^114^ NO at LiF(001),^111^ CO at Au(111),^73,113,115,116^ H_2_ at Ag(111),^106^ and HCl at Au(111)^117–119^ (see also the perspective paper^120^ and review^121^). For modelling of the collisional relaxation of highly vibrationally excited molecules, the energy landscape close to the dissociation barrier is very sensitive to the choice of the functional and strongly affects the predicted outcomes.^113^ The Born–Oppenheimer molecular dynamics (BOMD) simulations performed on the new NN-PES revealed a surprisingly large contribution to relaxation from an adiabatic mechanism for NO and CO scattering, arising from the softening of the vibrational potential for high vibrational states.^113,114^

Existence of high-dimensional accurate PESs initiated a new wave in MD simulations of diatomic molecules scattering from metal surfaces aiming to overcome the deficiencies of the previous approaches. Recent research carried out by theory groups of Maite Alducin (San Sebastian, Spain), Hua Guo (Albuquerque, New Mexico, USA), Bin Jiang (Hefei, China), Geert-Jan Kroes and Jörg Meyer (Leiden, The Netherlands), Reinhard Maurer (Warwick, UK), Joseph E. Subotnik (Philadelphia, Pensilvania, USA), and Jean Christophe Tremblay (Metz, France) brought a great deal of improvement in our understanding of the non-adiabatic dynamics at surfaces and was reviewed in detail in ref. 22, 103, 120, 122 and 123. Here, we focus on the results relevant for the topic of this work.

New BOMD simulations of NO (*v*_i_ = 3) scattering from Au(111) with an incidence energy of 1 eV performed on a NN-PES reproduced the translational energy distribution of scattered NO molecules seen in experiment.^69^ The experimental branching ratios, surface temperature dependencies for the vibrationally elastic and inelastic channels, were reproduced semi-quantitatively – see Fig. 16.

Interestingly, the translational inelasticity predicted for NO (*v*_i_ = 3 → *v*_f_ = 3) scattering from Au(111) was found to be independent of the way the electronic nonadiabaticity was accounted for and appears to be dependent only on the electronically adiabatic PES. The NN-PES proposed by Bing Jiang and coworkers^69^ leads to nearly perfect agreement with the experimentally observed translational energy distribution of the scattered NO (*v*_i_ = 3) (Fig. 16a). As suggested by Golibrzuch *et al.*,^62^ having an accurate adiabatic PES is essential for correctly capturing the incident translational energy dependence of vibrational relaxation probability. Indeed, the work of Bin Jiang and coworkers^69^ clearly demonstrates that more accurate NN-PES even in BOMD implementation is in qualitative agreement with the experiment^62^ where NO (*v* = 3 → 2, 1) relaxation probability was observed to grow with the increase of *E*_i_ – Fig. 16b and c. The bounce analysis of the scattered trajectories showed that the fraction of multi-bounce trajectories was much smaller with the new NN-PES,^62^ being clearly more accurate than the original one.^109^ Moreover, the NO steric effect observed in the experiment was also qualitatively reproduced.^112^ At the present state of the theory, the calculations of NO (*v* = 3 → 2, 1)/Au(111) vibrational relaxation^69^ on the new NN-PES were implemented with both BOMD and LDFA-based electronic friction model. LDFA-based EF resulted in very minor nonadiabatic vibrational energy transfer, very similar to BOMD (shown in Fig. 16b and c), both underpredicting the magnitude of vibrational relaxation. While more accurate NN-PES is an important advancement in correctly capturing the trends in vibrational relaxation, it must be complemented by more advanced approaches to describe electronically nonadiabatic vibrational energy transfer.

These calculations reveal a much more significant role of the adiabatic channel in vibrational relaxation of NO (*v*_i_ = 3) than was previously realized. Compare, for example, the experimentally observed values (black filled squares) to BOMD prediction (red triangles) in Fig. 16b – the contribution of adiabatic vibrational relaxation can be as high as ∼50% at *E*_i_ ∼ 1 eV. Similarly, the BOMD simulations of the NO (*v* = 16) scattering from Au(111) on the same NN-PES revealed rather unexpectedly large contribution of adiabatic vibrational relaxation, which was attributed to the softening of the potential in the neighborhood of the dissociation barrier. Including electronic friction at the level of LDFA did not, however, affect the outcome of simulations, whereas the use of ODF friction showed enhanced nonadiabatic vibrational energy losses.^110,111^

Similar conclusions about the importance of an accurate adiabatic PES were derived from modelling the CO (*v* = 17) and CO (*v* = 2) scattering from Au(111) using the IESH approach with diabatic PESs derived from the constrained DFT and represented by a high-dimensional neural network.^116^ Angular distributions, vibrational branching ratios and the influence of incidence energy on mean scattering translational energy agree quite well with those obtained from experiment.

We emphasize here that an accurate high-dimensional PES is a necessary but not sufficient ingredient of the modelling of molecular scattering from metal surfaces. Another important component is the propagation method accounting for the non-adiabatic effects. One of the evident deficiencies of both the MDEF and IESH approaches is that they treat nuclear degrees of freedom classically, which is probably valid for translational degrees of freedom, but may not be for rotational and vibrational degrees of freedom. As the full-dimensional nuclear quantum dynamics of molecular scattering is yet a formidable task, it would be highly desirable to estimate how important these quantum effects might be. One promising approach within this context is the method of stochastic wave-packet dynamics with dissipative rates obtained from Fermi's Golden Rule.^124,125^ The approach was applied for NO (*v*_i_ = 3) scattering from Au(111) qualitatively reproducing the vibrational relaxation probability dependence on incidence energy. Another interesting insight was provided by the recent work of Malpathak and Ananth,^126^ where the authors employed linearized stationary-phase path-integral dynamics capable of accounting for the zero-point energy and tunneling effects in NO scattering from Au(111). They used Hamiltonian (3) to model the electronic degrees of freedom. The diabatic PES and coupling were represented in terms of a 2D – NO bond length and its distance from the surface – model, developed for comparing different non-adiabatic propagators.^127^ The authors found that their semi-classical approach supports the experimentally and theoretically well-established conclusion on the dominant role of the charge transfer in the NO vibrational energy losses.

Scattering of the HCl molecule from Au(111) was also modeled by BOMD on the high-dimensional NN-PES.^117^ The simulations were able to explain qualitatively large translational energy losses and the dependence of the vibrational excitation on the incidence energy, though the translational and vibrational energy of the scattered molecules was much higher than in the experiment. Training NN-PES on the different DFT functional allowed one to somewhat improve the results but the agreement between theory and experiment remained unsatisfactory.^119^ Accounting for nonadiabatic effects with LDFA displayed only minor improvement.^118^ This led to the assertion that more advanced methods accounting for nonadiabaticity are required for this system.^120^

While theory has made significant advances in its ability to describe the nonadiabatic effects observed in experiment, there remain unresolved issues.

## 5. Unresolved issues

### 5.1 Electronically nonadiabatic *V*–*T* and *T*–*V* energy transfer: is *T* a spectator DOF?

**Fig. 17:**
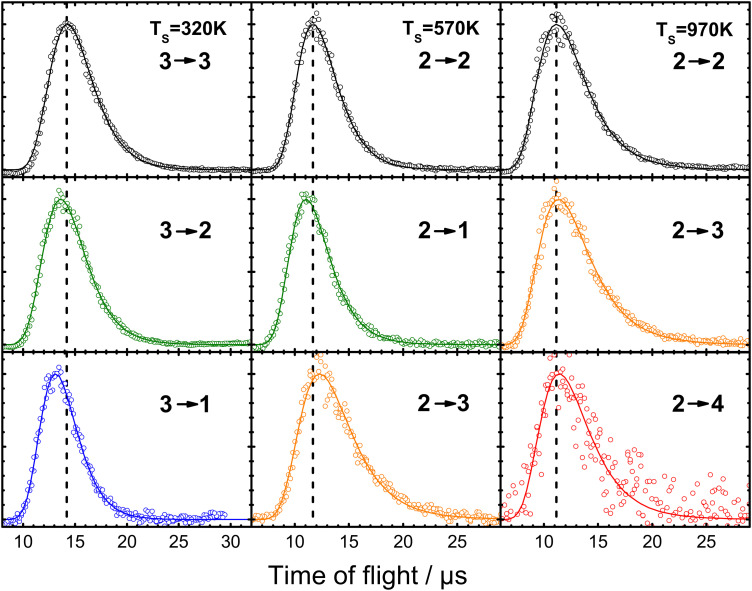
Example of experiments where laser-prepared NO (*v*_i_, *J*_i_) with well-defined translational energy of incidence collides with a Au(111) surface within a narrow arrival time window. The resulting (quantum-state-resolved) NO (*v*_f_, *J*_f_) time-of-flight traces indicating the time it takes to recoil from the surface to the (REMPI) detector are recorded. The vertical dashed lines are drawn to the peaks of the time-of-flight distributions of the elastic channels to illustrate the small differences for the vibrationally inelastic channels. The data cover vibrationally elastic scattering (black) as well as loss of one (green) and two (blue) quanta and gain of one (orange) and two (red) quanta of molecular vibration. From ref. 39 with permission from American Chemical Society, Copyright (2013).

The primary concern of theory so far has been to capture properly the exchange of energy between molecular vibration and the metal's EHPs. However, when a molecule collides with a metal surface, things are more complex; energy may be exchanged between the solid's EHP and phonon energy reservoirs and the molecule's translational, vibrational, and rotational DOFs. Fig. 17 shows an example of some of this complexity.^39^

This figure shows results on the NO (*v* = 2 → 1, 2, 3, 4) and NO (*v* = 3 → 3, 2, 1) scattering channels for collisions with Au(111)^39^ at a broad range of surface temperatures, 320 K < *T*_S_ < 970 K. They clearly demonstrate that the spectator view of the translational motion in electronically non-adiabatic vibrational energy transfer is only approximately correct. It is immediately apparent from the raw time-of-flight data illustrated in Fig. 17 that vibrationally inelastic channels exhibit different scattering velocities than vibrationally elastic ones. This indicates that some fraction of the released vibrational energy is channeled to outbound translation, while some fraction of incident translational energy is channeled to vibrational excitation. This work provides a useful extension of prior work, where *V*–*T* coupling was reported for the HCl (*v* = 2 → 1)/Au(111) channel at only a single surface temperature. Here, ∼26% of the released vibrational energy appeared as HCl translation.^128^

**Fig. 18:**
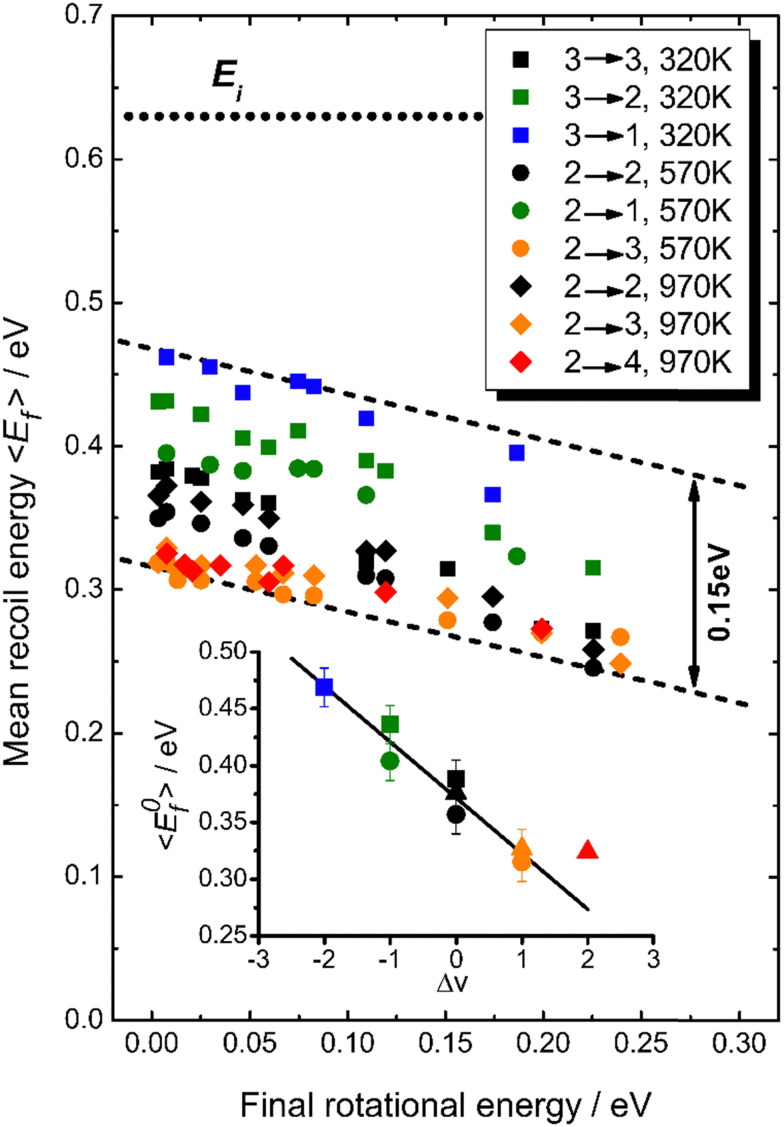
*T*_S_-Corrected mean recoil energies *vs.* final rotational energies (all values are shifted to *T*_S_ = 570 K). The dotted line indicates the incidence energy of 0.63 eV. While the range of vibrational energy change covers −0.47 eV ≤ Δ*E*_vib_ ≤ 0.47 eV, the variation in translational energy only varies over 0.15 eV, as indicated by the dashed lines. The inset shows the *T*_S_-corrected recoil energies extrapolated to zero rotational energy as a function of the change in vibrational quantum number, Δ*v*. A linear fit yields a slope of −0.049 eV per vibrational quantum, corresponding to ∼21% of vibrational energy coupling to translation (the value for Δ*v* = +2 corresponding to noisy data was excluded from this fit). From ref. 39 with permission from American Chemical Society, Copyright (2013).

Fig. 18 summarizes observations of *V*–*T*/*T*–*V* energy transfer observed in vibrational relaxation/excitation events occurring in NO collisions with Au(111).^39^ The magnitude of the vibrational energy exchanged in these experiments spans the range from −0.47 eV to +0.47 eV, corresponding to losing/gaining two vibrational quanta. By contrast the variation in translational inelasticity spans the range of about 0.15 eV. After correcting the recoiled 〈*E*_f_〉 by surface temperature and rotational energy dependence, it is found that ∼21% of the vibrational energy exchanged is converted to or taken from translation.^39^ This is similar to the HCl/Au(111) system reported in ref. 128. Both of those results were for collision energies of about 0.6 eV. At incidence energies of 1 eV, the *V*–*T* coupling was even larger ∼34%.^61^

The origin of *V*–*T*/*T*–*V* coupling in electronically nonadiabatic vibrational energy transfer remains unclear. This has not prevented speculation^39^ about (1) acceleration of the transient NO^−^ anion towards the surface due to the image charge force, (2) surface site specific enhancement of electronically nonadiabatic coupling, (3) a neglect of accounting for adiabatic coupling between translation and vibration and (4) EHP mediated coupling between translation and vibration.

Theoretical descriptions of these subtle, yet important phenomena are still inadequate; however, several theoretical groups are currently developing accurate adiabatic PESs along with advanced approaches to EHP-V coupling that promise improvements.

### 5.2 Translational–rotational anticorrelation

**Fig. 19:**
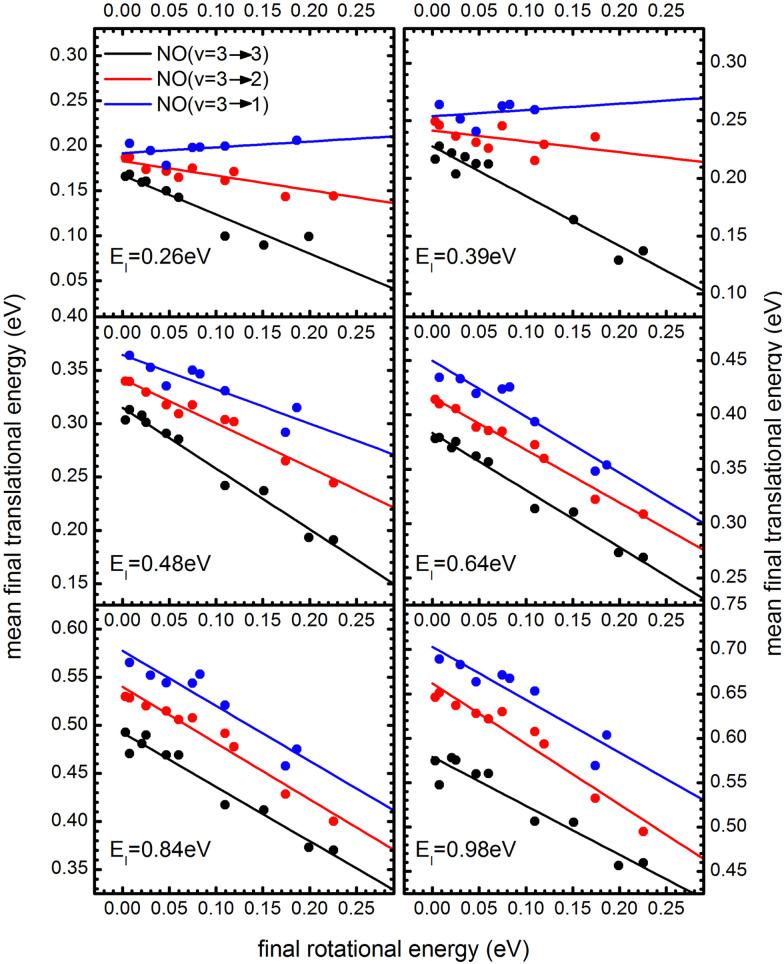
*T*–*R* correlations and anticorrelations in NO scattering from Au(111). 〈*E*_f_〉 *vs. E*_rot_ for NO (3 → 3, 2, 1)/Au(111) at various translational energies of incidence. For all cases, the so called “*T*–*R* anticorrelation” is observed manifested in 
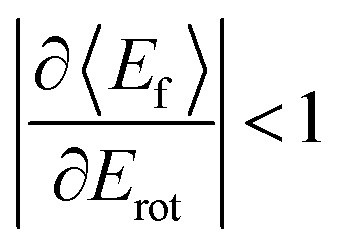
. The deviations from unity are more pronounced for vibrationally inelastic channels, especially at low values of incident translational energy. From ref. 61 with permission from the PCCP Owner Societies Copyright (2014).


The coupling between translational and rotational degrees of freedom deserves a special note – see Fig. 19.^61^ It shows the observed (approximately linear) correlation between mean final translational 〈*E*_f_〉 and rotational energy *E*_rot_ of the NO molecules scattered from Au(111) for three vibrational channels *v*_f_ = 3, 2, 1 for six incidence energies of translation. The physical meaning of the slope of 〈*E*_f_〉 *vs. E*_rot_ can be understood from two limiting cases.

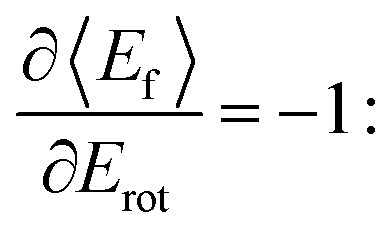
 all energy released in the collision appears as either translation or rotation.


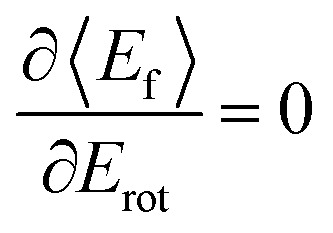
: anti-correlation between outbound translation and rotational excitation.

For nearly all cases shown in Fig. 19, 
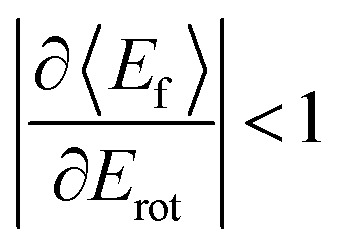
 is observed. Deviations from this behavior become more pronounced at reduced incidence energy, especially for vibrationally inelastic scattering channels.

Similar results were obtained for NO (*v* = 0 → 0)/Ag(111) scattering^129,130^ and were explained by a simple kinematic model only taking into account the orientation of the molecule at impact, the initial kinetic energy and the masses of the atoms. In detail, when the molecule collides with the surface, a part of the initial translational energy is transferred to the surface phonons and another part is transferred to molecule's rotation. The distribution between these two DOFs depends on the orientation of the molecule at impact: those orientations that lead to large rotational excitation also lead to small energy transfer to phonons. Consequently, the translation–rotation coupling as well as the scattered rotational state distribution is a measure of the molecule's orientation when it hits the surface. It is likely that collisions with “N-down” orientations of the molecule, which are characterized by an attractive well in the PES, lead to more energy transferred to phonons. Such collisions may also exhibit little rotational excitation, as this is the stable orientation of the molecule. On the other hand, collisions with “O-down” (unstable) orientations may result in less translational energy being transferred to surface phonons and a greater degree of rotational excitation. Similar *T*–*R* anticorrelation effects, attributed to strongly orientation-dependent attractive potential, causing the NO molecule approaching the surface with the O-end to experience higher rotational excitation, were reported for NO (*v* = 0 → 0)/Ge scattering.^131^ Since such “dynamical steering” effects are more pronounced at low translational energies of incidence, we might expect a decrease of the slope in *T*–*R* correlation plots with decreasing *E*_I_. These findings are consistent with the observations of “direct” orientation experiments, highlighted in Section 3.7.

Since vibrational relaxation is favored when the N-atom is oriented towards the Au(111) surface, this might also be the reason for observation of enhanced *T*–*R* anticorrelation 
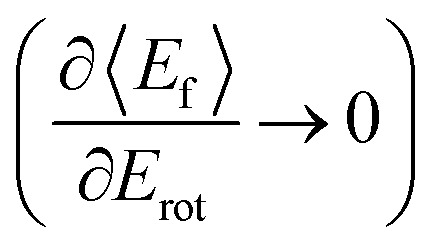
 for vibrationally inelastic scattering channels at low incidence energy.^61^ Another possible explanation for the enhanced “flattening” of the *T*–*R* correlation slope in vibrationally inelastic events can originate from the fact that inelastic channels are more sensitive to multi-bounce collisions, which scramble the translation–rotation coupling.

In summary, at present the dynamics of *V*–*T*/*T*–*V* transfer and *T*–*R* (anti)correlation in electronically non-adiabatic vibrational energy transfer remain incompletely understood. It remains to be seen if new accurate PESs can more accurately predict multibounce/single bounce collisions and perhaps yield a quantitative description of the subtle *T*–*V* and *T*–*R* observations accompanying nonadiabatic vibrational energy transfer.

## 6. Summary and outlook

In a perspective article devoted to quantification of Born–Oppenheimer approximation (BOA) breakdown in vibrational energy transfer on metal surfaces, published more than a decade ago,^3^ experiments were still highly challenging and observations were still quite limited. Thirteen years down the road, many of the original “miracle experiments” have become routine and a comprehensive array of experimental observations characterizing BOA breakdown at a level of detail that is rare in chemical physics has become available. An intuitive unifying model has emerged, based on electron transfer driven vibrational energy transfer that is capable of explaining, at least on a semi-quantitative level, the vast body of experimental observations, when implemented within the modern nonadiabatic theoretical framework.

Despite these successes, our knowledge is still derived from a rather small number of systems; for example, data derived from NO and CO scattering from Ag(111) and Au(111) make up the lion's share of our observational knowledge-base. Future studies to expand observations to other systems, in particular to those that can react under conditions where nonadiabatic behavior might be expected, are highly desirable. An obvious extension of existing work in this direction would involve O_2_ interactions with metal surfaces. Due to its high electron affinity, we expect electron transfer dynamics to be at work for this molecule when it collides at many metals. Due to its weaker bond, it is not hard to find metals where O_2_ is likely to undergo dissociative adsorption. Spectroscopic methods for O_2_ are subject to the consequences of rapid predissociation in excited electronic states – these consequences must be dealt with for example with picosecond SEP or employing new REMPI detection schemes.^132^

The interactions of NO with Cu are also interesting as dissociation is also likely here. As the lightest coinage metal, copper's higher reactivity than silver and gold led early experiments to focus on NO interactions only with oxidized copper due to the difficulty in maintaining clean copper under molecular beam exposure.^133^ Recent experimental advancements suggest potential resolutions to these challenges^46^ raising the possibility of quantum state-resolved experiments on reacting NO on clean Cu(111). A promising and fascinating experiment would probe vibrational quantum state dependent surface reactivity, which may be strongly influenced by nonadiabatic effects. Ultimately, we would like to know if there are reactions taking place at metal surfaces, whose rates cannot be computed within the BOA.

Overall, experimentalists have generated a huge amount of detailed data over the past decade that serve as a perfect benchmark for testing of high-level *ab initio* theory that goes beyond the BOA. The recent theoretical studies on diatomics scattering sketched out in Section 4 showed that the mechanism where vibrational relaxation is only due to strong coupling EHPs as was suggested earlier appears to be a drastic oversimplification. One has to take into account an interplay of weak coupling electronic friction-like mechanisms, V-EHP strong coupling and purely adiabatic energy transfer. Their relative importance depends on incidence energy and angle, initial vibrational and rotational excitation, molecular alignment and the electronic structure of a molecule and surface. While we have unquestionably made dramatic experimental advances, some of the most essential experimental observations still await theoretical treatment. In light of recent improvements to theory, we may be optimistic that these theoretical breakthroughs will soon be realized.

## Conflicts of interest

There are no conflicts of interest to declare.

## Supplementary Material
